# Biomedical literature classification with a CNNs-based hybrid learning network

**DOI:** 10.1371/journal.pone.0197933

**Published:** 2018-07-26

**Authors:** Yan Yan, Xu-Cheng Yin, Chun Yang, Sujian Li, Bo-Wen Zhang

**Affiliations:** 1 Department of Computer Science and Technology, School of Mechanical Electronic and Information Engineering, China University of Mining and Technology, Beijing, 100083, China; 2 Department of Computer Science and Technology, School of Computer and Communication Engineering, University of Science and Technology Beijing, Beijing 100083, China; 3 Key Laboratory of Computational Linguistics, Peking University, Ministry of Education, Beijing 100871, China; City University London, UNITED KINGDOM

## Abstract

Deep learning techniques, e.g., Convolutional Neural Networks (CNNs), have been explosively applied to the research in the fields of information retrieval and natural language processing. However, few research efforts have addressed semantic indexing with deep learning. The use of semantic indexing in the biomedical literature has been limited for several reasons. For instance, MEDLINE citations contain a large number of semantic labels from automatically annotated MeSH terms, and for a great deal of the literature, only the information of the title and the abstract is readily available. In this paper, we propose a Boltzmann Convolutional neural network framework (B-CNN) for biomedicine semantic indexing. In our hybrid learning framework, the CNN can adaptively deal with features of documents that have sequence relationships, and can capture context information accordingly; the Deep Boltzmann Machine (DBM) merges global (the entity in each document) and local information through its training with undirected connections. Additionally, we have designed a hierarchical coarse to fine style indexing structure for learning and classifying documents, and a novel feature extension approach with word sequence embedding and Wikipedia categorization. Comparative experiments were conducted for semantic indexing of biomedical abstract documents; these experiments verified the encouraged performance of our B-CNN model.

## 1 Introduction

With the rapid development of biomedicine, there has been an increase in the amount of biomedical literature. Semantic indexing is vital for biomedicine document classification and retrieval. Semantic indexing for biomedical documents is multi-label classification problem to assign the articles with controlled vocabulary thesaurus, MeSH (Medical Subject Heading) terms. Table 1 shows an example of a biomedical article with manually annotated MeSH terms. The annotation work would consume immense manpower and financial resources. The support for retrieving newly-published articles would also be delayed. The main purpose of the task is to automatically classify new PubMed documents and approximate the results with the manually annotated classes from MeSH headings provided by Pubmed curators. As illustrated, the original text of article title as well as abstract are given. Over 20 thousand descriptors in MeSH headings are provided as candidates of which one or some are associated with the article. There are two major research focuses with respect to semantic indexing [1–3]. The first is based on shallow learning approaches, which usually contain a process of matching correlated documents by comparing keywords. This is the most primitive and direct method. The other is based on deep neural networks (DNNs) [4], which have emerged as a powerful machine learning technology. DNNs have been widely applied and proved to be effective in image classification [5–7], speech recognition [8–10], and natural language processing (NLP) tasks [11–13]. Through several comparisons, they are proved to have significant advantages compared to state-of-the-art shallow learning methods for semantic indexing [14–16].

**Table 1:** An example PubMed article with manually annotated MeSH terms.

Journal: Photochemistry and photobiology
Year: 1983
Title: Kinetics of bacterial bioluminescence and the fluorescent transient.
Abstract: The addition of FMNH(2), to Vibrio harveyi luciferase at 2°*C* in the presence of tetradecanal results in the formation of a highly fluorescent transient species with aspectral distribution indistinguishable from that of the bioluminescence. The bioluminescence reachesmaximum intensity in 1.5 s and decays in a complex manner with exponential componentsof 10(-1) s(-1), 7 x 10(-3)S(-1). and 7 x10(4)s(-1). The fluorescent transient rises exponentially at7 x 10(-2)s(-1) and decays at 3 x 10 (4)s(-1). The slowest bioluminescence component. comprising the bulk of the bioluminescence. decays at twice the rate of the fluorescent transient under all variations of reaction conditions: concentration of reactants. temperature 2–20°*C*. and aldehyde chain length - decana1, dodecanal and tetradecanal. The activation energy for both the slowest bioluminescence decay and the transient fluorescence decay is 80 kJ-mol(-1). An energy transfer scheme is proposed to explain the results where two distinct chemically energized species utilize the fluorescent transient as emitter for the slower bioluminescences, and for the faster process a fluorophore present in the protein preparation. Kinetic observations suggest that typical preparations of V. harveyi luciferase comprise 15% active protein.
MeSH terms: “Flavin Mononucleotide” “Fluorescence” “Kinetics” “Luciferases Luminescence” “Time Factors” “Vibrio”

In 2011, researchers from Microsoft Research and Google used DNN technology for speech recognition to decrease the error rate of 20% -30% [14]. In 2012, DNN technology was utilized on ImageNet task [16], which reduce the error rate from 26% to 15%.

In the field of biomedicine, MeSH indexing, the mostly applied mainstream method, mines the context of indexed PubMed and MEDLINE citations [17–21] based on the bag-of-words model (BOW). Nowadays, several statistical models and language models in information retrieval and text mining are introduced to combine with MeSH mapping [22–25]. However, this type of feature representation only contains information related to word frequency. In many common area cases like sentiment classification, deep learning has been effectively used to represent more semantic information other than frequency. However, in biomedicine, conventional deep learning methods might face some more challenges, such as terminologies, synonyms based on professional knowledge [26, 27]. The MeSH term “Luciferases Luminescence” in the example within Table 1 cannot be easily mapped without professional knowledge. Indeed, indexing is hindered by a large quantity of professional terms, a lack of information (generally only titles and abstracts, which may cause data sparsity for learning), and a high correlation between different labels. Hence, few research efforts have addressed for indexing with deep learning.

Convolutional neural networks (CNNs) [28, 29] provide a flexible framework that can be used to reduce variation and exploit spatial correlations using weight sharing and local connectivity. However, the sizes of the sliding windows of the convolutional kernels can be difficult to determine. Large windows can hinder training because they necessitate an enormous number of parameters [30]. Conversely, small window sizes may lead to the loss of some critical information, which then needs to be estimated. The deep Boltzmann machine (DBM) [31] represents a good feature extraction method in deep learning, as it can be used to effectively combine global and local information. In this paper, taking biomedical abstract semantic indexing as a case study, we propose a hierarchical CNNs-based (coarse to fine) indexing framework and present a suitable loss function for handling specific domain terms and the correlation of labels for hierarchical indexing of biomedical documents. As indexing must be accomplished using only the title and abstract information of documents, we used word sequence embedding together with Wikipedia categories and entity classes to enrich document representation. Our empirical results verify that this improved representation is denser than that obtained via the BOW model.

To summarize, this paper represents three major contributions to the semantic indexing literature. First, to the best of our knowledge, we present the first case study of biomedical document semantic indexing with CNNs, including comparisons with several state-of-the-art methods. Second, we offer a framework for combining CNNs with DBM to learn document representation, where CNNs extract local information regarding the context and the DBM is used to merge the global features. We use a suitable loss function for the training of this framework, where multi-label classification is performed in a coarse-to-fine learning style. Third, we propose a new way to enrich document representation using sequence information.

This paper is organized as follows. Related work is described in Section 2. In Section 3, we describe the proposed method for semantic indexing with CNNs. In Section 4, we describe semantic feature extension for a document. We demonstrate comparative experiments in Section 5 and present final remarks in Section 6.

## 2 Related work

### 2.1 Semantic indexing for documents

The existing semantic indexing approaches can be divided into two main categories, according to the semantic representation methods. One group of methods tend to represent semantics with shallow artificial features, like the BOW and term frequency-inverse document frequency (TF-IDF) models, which only contain information about word frequency. However in most cases, the corresponding lexical matching would cause missing results because of different expressions of significant concepts, for example aliases, synonyms or abbreviations. Several learning methods have been proposed with attempt based on the shallow semantic features, such as latent semantic indexing (LSI), latent Dirichlet allocation (LDA) and probabilistic latent semantic indexing (pLSI) [32–34]. LSI aims at decomposing the feature matrices with SVD (Singular Value Decomposition), to select a subset of the original features to represent the semantics. The process is similar to dimension reduction with Principle Component Analysis. Through the removal process, which is implemented by assigning weights to 0, the unimportant latent semantic features are filtered out. A vital weakness of this method is the inability of processing the problem of polysemy. pLSI constructs a semantic representation model in which each document uses massive parameters. Consequently, when processing large number of documents, the overfitting problem are unavoided. In order to solve the above limitation, LDA was proposed to eliminate the dependency between the number of parameters in the model and the number of documents. However, most of these above models are linear models with unsupervised dimension reduction [35], and are based on the unordered hypothesis (exchangeability). That is, the sequence of the words in the document is always ignored; thus, the semantic representation is inappropriate for some sequences [3]. Recently, some researchers have attempted to apply supervised learning techniques to optimize the semantic representation of documents. Typically, supervised LDA [36] adds an extra response variable to LDA by generalizing linear models with respect to the EM (Expectation Maximization) algorithm associated with each document. However, the query and the document are regarded as independent and processed separately in the learning process. Supervised semantic indexing [37] focuses on the correlations between words in queries and candidate documents, and learning to rank algorithms are then utilized to select the best combination of features from a large feature set generated from all word pairs.

In the last several years, deep learning has arisen the focus of all researchers in the research field of artificial intelligence, with a variety applications on image classification [16], object recognition [38, 39], speech recognition [14], and NLP tasks [15, 40]. Like other applications, there are some investigations about trying deep learning techniques for semantic indexing. Salakhutdinov and Hinton [1] proposed a novel representation method for extending semantic indexing. An deep auto-encoder model was proposed in that work with the combination of a higher layer encoded with binary codes and a lower layer generated based on word frequency vectors. The constrained Poisson model was also introduced to deal with documents with various lengths. Another work was proposed by Mirowski [2] to improve the deep auto-encoder model by introducing a dynamic variable. This variable is generated from a gradient-based MAP (Maximum a Posteriori) inference and can help to compute the encoder and the decoder. With an additional use of the document label, the classifiers can be successfully trained. Socher [41] proposed a semantic indexing method with the recursive neural network, which captures the semantics of sentences via a tree structure for labeled structure prediction. This model has been found to be efficient for constructing sentence representations when mapping to images. Wu [3] designed a deep structure with restricted Boltzmann machines (RBMs) to compute semantic representation of documents. This model was improved in [42] which develops the feature space can make labels less interdependent and easier to model and predict at inference time. CNN was firstly utilized in semantic indexing in [43] with dynamic max pooling and hidden bottleneck layers. There are also several studies working for semantic indexing for images, videos, etc [44–46]. Through experiments in the above papers, deep semantic embedding models with nonlinear features can grasp the semantics with more accurate and robust representations. These models also use discriminative fine-tuning, and improve the ranking of documents with more appropriate scoring functions. Indeed, deep models are associated with an increase in indexing performances.

### 2.2 Biomedicine document classification

Several studies have been investigated natural language processing techniques in the health domain [47–49]. MTI (Medical Text Indexer) [20] is a document indexing system that provides recommendations based on the Medical Subject Headings (MeSH) and MEDLINE databases. MTI has two main components: MetaMap Indexing and PubMed Related Citations (PRC). The MetaMap is a module that maps biomedicine documents to concepts in the UMLS Metathesaurus. The PRC algorithm is a modified kNN algorithm that relies on document similarity to assign MeSH headings. The results produced by the two paths are given weights via post-processing (such as clustering and merging), and these can be used to retrieve a ranked list of MeSH terms.

The MeSHUP system [21] explores the combination of different machine learning approaches for classification of full class-sets. This system is highly scalable and capable of improving biomedical IR from a ranked output of MeSH terms.

### 2.3 CNNs for document classification

Lai [30] proposed the recurrent convolutional neural networks (RCNN) model for document classification, with the goal of overcoming the Recurrent Neural Network bias whereby later words are more dominant than earlier words. The RCNN effectively utilizes the advantages of the recurrent structure, which captures the contextual information, and learns the feature representation of documents using CNN. Shen [50] proposed a new latent semantic representation for web searches based on CNN. The word n-gram (word sequence) information is set as the local feature and is calculated via convolution and max pooling to learn the high-level semantic representation. Johnson [51] explored the application of CNN for converting images (2D structure) to text (1D structure). In documents, the word order is entered as a 1D structure, and each word representation uses a vector based on the BOW. Experiments indicated that this model achieved good performance with respect to sentiment classification and topic classification. Santos [26] proposed a deep convolutional neural networks model using character-level, word-level, and sentence-level features for sentiment analysis of short texts. These features are not based on handcrafted inputs but on word-level embedding. Additionally, these features, which are operated by a convolutional layer, are able to capture the global feature vectors of sentences and extract the relevant features from any part of a word.

## 3 Model

**Fig 1:**
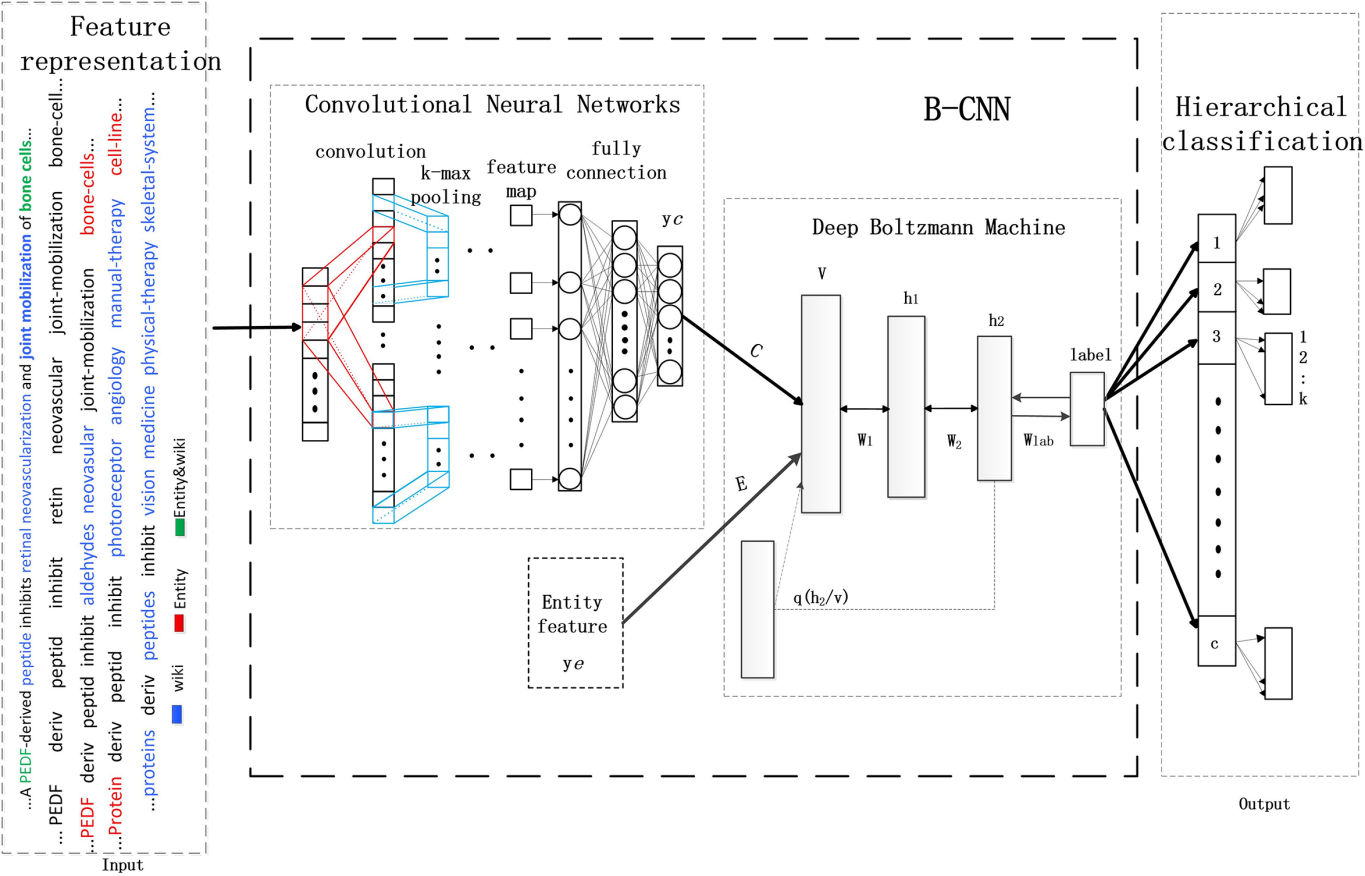
CNNs-based hybrid learning framework.

Considering the numerous classes of documents and the imbalance in the distributed samples, we developed a framework in which hierarchical CNNs were combined with DBMs. This framework can perform multi-class and multi-label semantic indexing with correlated labels for biomedicine documents (we call this B-CNN). The architecture of our proposed framework is shown in Fig 1.

The model consists of three parts, i.e., feature representation (the input of the framework), the B-CNN model, and multi-label hierarchical classification (the output of the framework). In this section, we mainly describe the B-CNN model and the components of hierarchical indexing. B-CNN combines the advantages of both CNNs and DBMs. CNNs are more suitable for capturing context information from features with sequence relationships. DBMs are advantageous in that they can merge global and local information through undirected connections, thus effectively removing noise from different representations. The hierarchical B-CNN based indexing is much better than flat classification for processing a large number of classes. Moreover, the coarse clustering step is an effective way to remove noise from unbalanced distributed samples. In addition, we designed suitable loss functions for the learning process of this framework. The details of these two parts are described as follows.

### 3.1 Convolutional neural networks

Since the error gradients and the BP (Back Propagation) algorithm into the CNNs to simlify the training process [28, 29], CNNs become more widely-used for feature representations. A simple CNN structure acting as sentence model is made up with one-dimensional convolutional layer followed by a k-max pooling layer (see the CNN model in a single Fig 1). Different from conventional neural networks, each neuron in convolutional layer is only connected with a local subset of the neurons in the lower layer. In this way, the neuron merely aims at extracting the local information of the corresponding lower layer. Each neuron represents local features from the connecting receptive field (which may or may not overlap), depending on the activation function within the neuron. As a result, convolution neurons play the role of feature detectors, and the weights of the neural connections decide the characteristics of the reaction (i.e., the degree of the reaction). Through feature detectors, several feature maps are generated by convolution kernels and the networks can effectively detect the local features at every position of the preceding layer. The parameters for each nodes within the same feature map are the same for they share a same convolution kernel [30]. Due to this characteristics, the number of parameters within the network can be eliminated to a large extent compared with conventional neural networks, which makes the structure simpler and more flexible. Max pooling layer is utilized for feature mapping, and the operator is a nonlinear subsampling function that returns the maximum of a set of values [40].

In each convolution layer, every input sample vector is weighted with several different convolutional kernels, and various degrees of local features are extracted by varying the sizes of sliding window. Following the feature representation layers(convolutional and pooling layers), there are fully-connected layers where take the each feature maps as input and the neurons are connected like conventional networks. Through a certain number of convolutional kernels, the semantic representations of documents can be easily enriched.

Assume the input sequence matrix is *A*, the kernels used for convolution and pooling are *K* and *β*. And $b_{i}^{1},b_{i}^{2}$ are respectively the bias vectors for convolutional layer and pooling layer. The output of convolutional layer, pooling layer and fully-connected layer are:

$$\begin{array}{c} a_{i}^{1}=f\left(conv2\left(A,K\right)+b_{i}^{1}\right) \end{array}$$ (1)

$$\begin{array}{c} a_{i}^{2}=f\left(\beta down\left(a_{i}^{1}\right)+b_{i}^{2}\right) \end{array}$$ (2)

$$\begin{array}{c} y_{c}=f\left(wa_{i}^{2}+b\right) \end{array}$$ (3)

where *conv*2 is the convolution of two matrices, *down* represents the sampling operation to implement max-pooling, $K,\beta ,b_{i}^{1},b_{i}^{2}$ and the parameters *w* in fully-connected neural networks make up with the parameter set of CNN.

### 3.2 Deep Boltzmann machines

**Fig 2:**
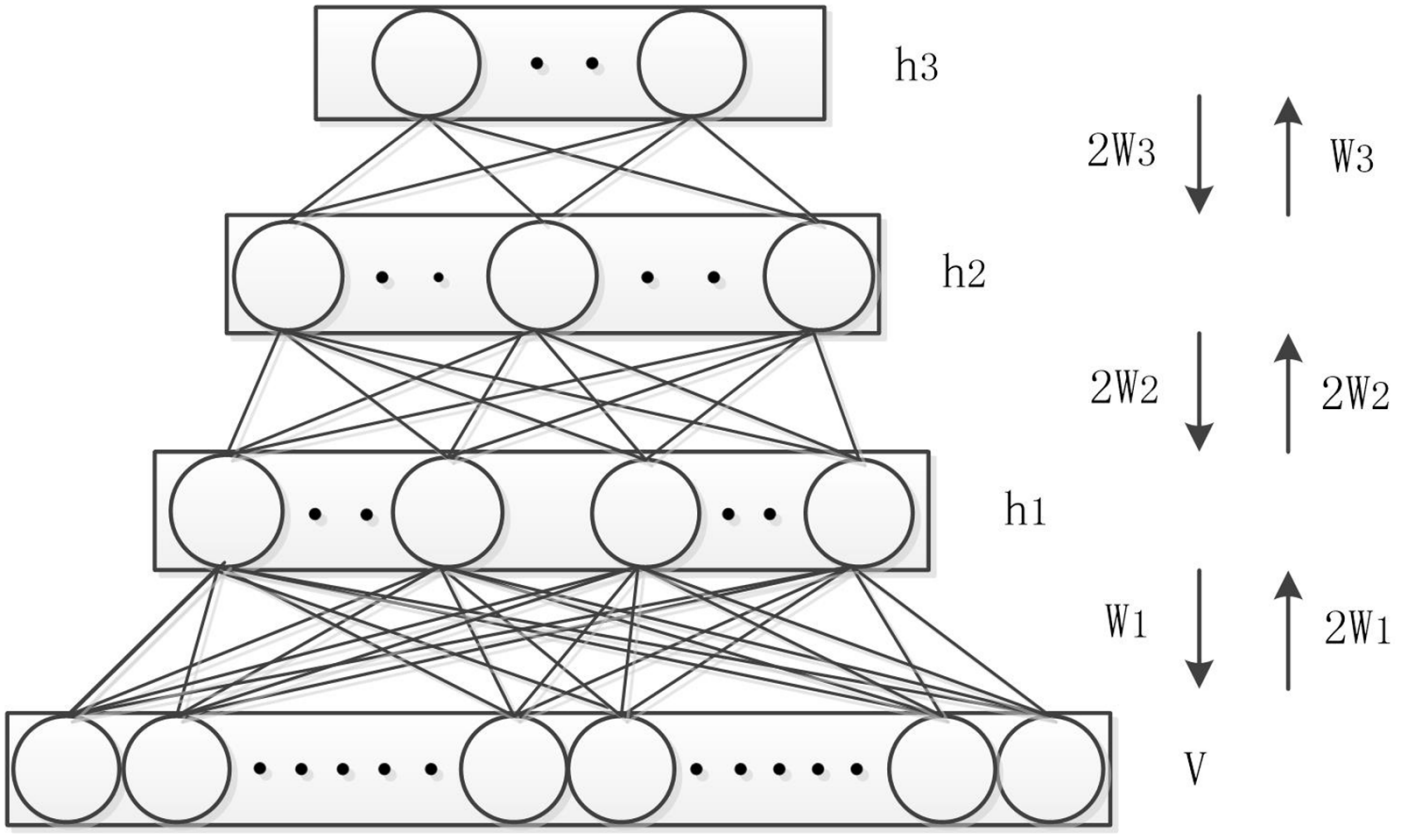
Deep Boltzmann machines.

The DBM [31, 52], which is composed of RBMs, is an undirected graph network of symmetrically stochastic binary units. The RBM [53] is a generated neural network that can learn probability distribution over a set of inputs. More specifically, an RBM is a kind of Boltzmann machine in which all visible and hidden units are fully-connected between layers and not connected within each layer. The visible units (***V***) represent the input of data, and the hidden units (***h***) represent features learned from the visible units. The DBM has many advantages: it retains and discovers layer presentations of the input with an efficient pertaining procedure; it can be trained on unlabeled data, and parameters of all layers can be optimized jointly in the likelihood function. Fig 2 shows how the layer nodes are constructed in the DBM. This is a three-layer network with no within-layer connections. ***h***_**1**_, ***h***_**2**_ and ***h***_**3**_ are three hidden layers with a different number of nodes for each level. The energy of the state {***V*,** ***h***_**1**_, ***h***_**2**_, ***h***_**3**_} is defined as:

$$\begin{array}{c} E(V,h_{1},h_{2},h_{3};\theta )=-V^{T}W_{1}h_{1}-h_{1}^{T}W_{2}h_{2}-h_{2}^{T}W_{3}h_{3}, \end{array}$$ (4)

where *θ* = {***W***_**1**_**, *W***_**2**_**, *W***_**3**_} are the model parameters, representing symmetric interaction terms.

During the process of calculating the value of the nodes in each layer, the undirected connection in each visible and hidden layers, which incorporates top-down feedback, plays a critical role in the performance of a DBM. This is the case for the DBM both as a generative and discriminative model. Additionally, this quality makes the feature representation of the DBM perform better than the deep belief network (DBN) [31].

### 3.3 Boltzmann-Convolutional neural network (B-CNN)

#### 3.3.1 Principle analysis

CNNs perform well when extracting local information about context. But for articles for which the abstract is the only available information, more global information may be required. Mikolov [54] showed that adding external context information can improve the performance of the model. The entity occupies an important position in the field of biomedicine indexing [55], so we combined the entity, as the complementary global feature information, with the input vector in each document.

The DBM, which updates weights using a priori knowledge learned from undirected graphs, represents a good feature extraction method in deep learning. Additionally, when analyzing the data structure of documents, we have found that the DBM can remove noise introduced by the document input. The DBM uses the nodes of both the former layer and the latter layer, as a result, hidden node sampling is more accurate. However, the DBM is disadvantageous in that the training time grows exponentially with the number of layers and nodes, as well as with the magnitude of the connection strengths [52]. To decrease the time complexity, we used a two-layer DBM, thus taking both training time and model accuracy into consideration for biomedicine indexing.

#### 3.3.2 Framework analysis

In Fig 1, the entity feature, which represents the global features of each document, is a vector with the same dimensions as ***y***_***c***_. The values (***V***) in the input layer of the DBM are computed as follows:

$$\begin{array}{c} V=f(Cy_{c}+Ey_{e}), \end{array}$$ (5)

where

$$\begin{array}{c} f\left(x\right)=\frac{1}{1+e^{-x}}. \end{array}$$ (6)

**C, E** are the weight matrices for which the likelihood of the training data is maximized. ***y***_***c***_ is the output of the CNNs model, and ***y***_***e***_ is the entity feature corresponding to each document.

**Fig 3:**
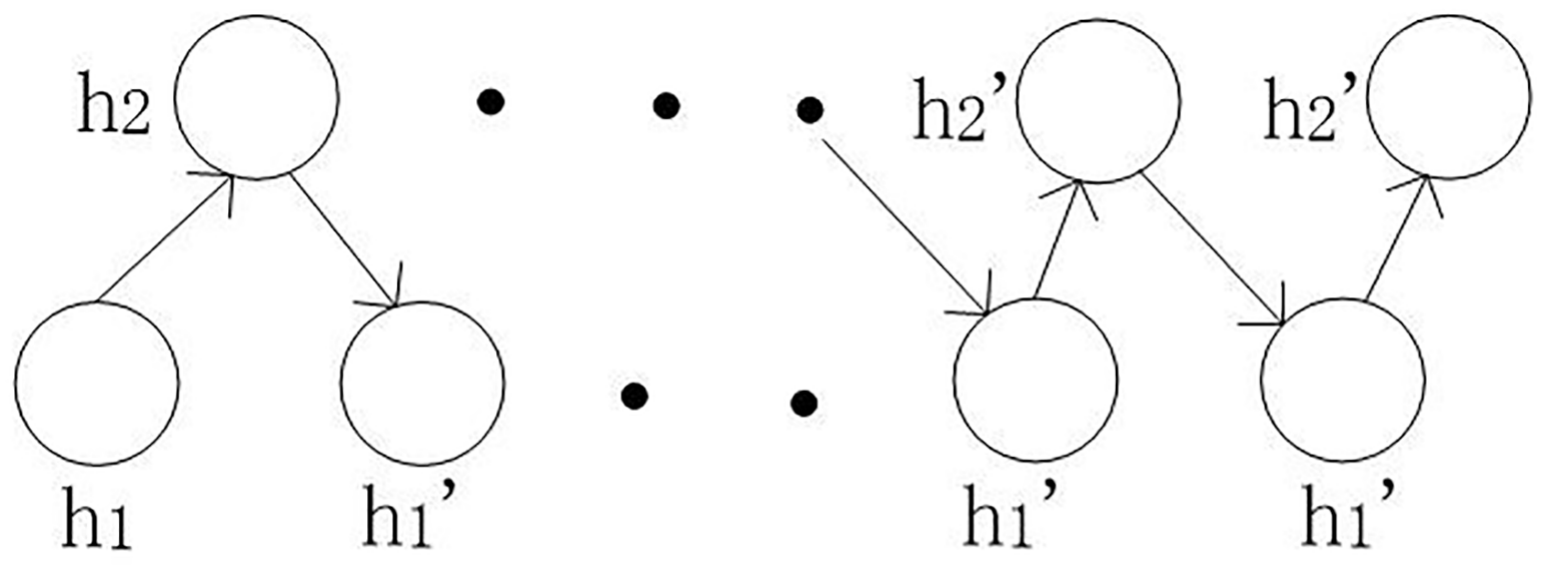
Layer’s nodes sampling process.

***W***_**1**_**, *W***_**2**_**, *W***_***lab***_ represent weight connections between the visible layer and the ***h***_**1**_ layer, ***h***_**1**_ layer and ***h***_**2**_ layer, and ***h***_**2**_ layer and the label layer, respectively. We calculated the values (***V*′** − ***V***) and ($h_{1}^{\prime }-h_{1}$), updating for weight. ***V*′** and ***h*′**, which are the reconstruction representations after each sampling (corresponding to ***V*** and ***h***, respectively), represent the state of the visible and hidden nodes, respectively. The process of sampling the nodes in the ***h***_**1**_ layer and ***h***_**2**_ layer is shown in Fig 3,

$$h_{1}\to h_{2}:\;h_{2}=h_{1}*W_{2}+tar*W_{lab}^{T}+bias1,$$ (7)

$$h_{2}\to h_{1}^{{}^{\prime }}:\;h_{1}^{{}^{\prime }}=V*W_{1}+h_{2}*W_{2}^{T}+bias2,$$ (8)

$$h_{1}^{{}^{\prime }}\to h_{2}^{{}^{\prime }}:\;h_{2}^{{}^{\prime }}=h_{1}^{{}^{\prime }}*W_{2}+tar*W_{lab}^{T}+bias3,$$ (9)

where *tar* represents the real label of the training sample, and *bias* is the bias value between the visible and hidden layers.

**Fig 4:**
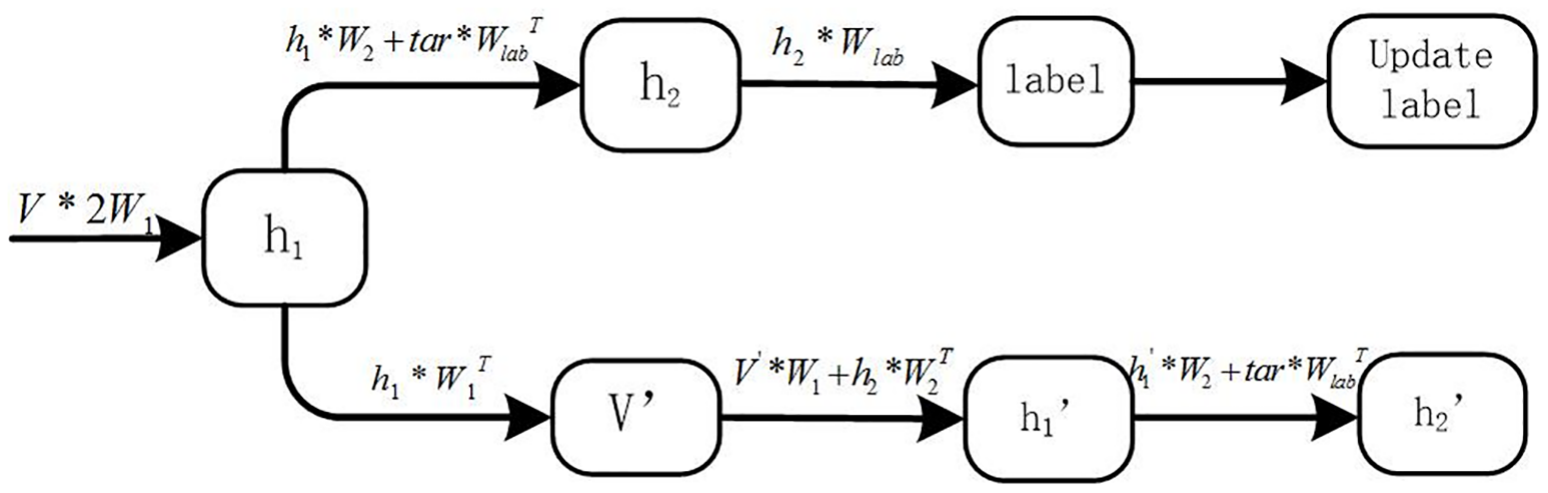
Training process.

**Fig 5:**
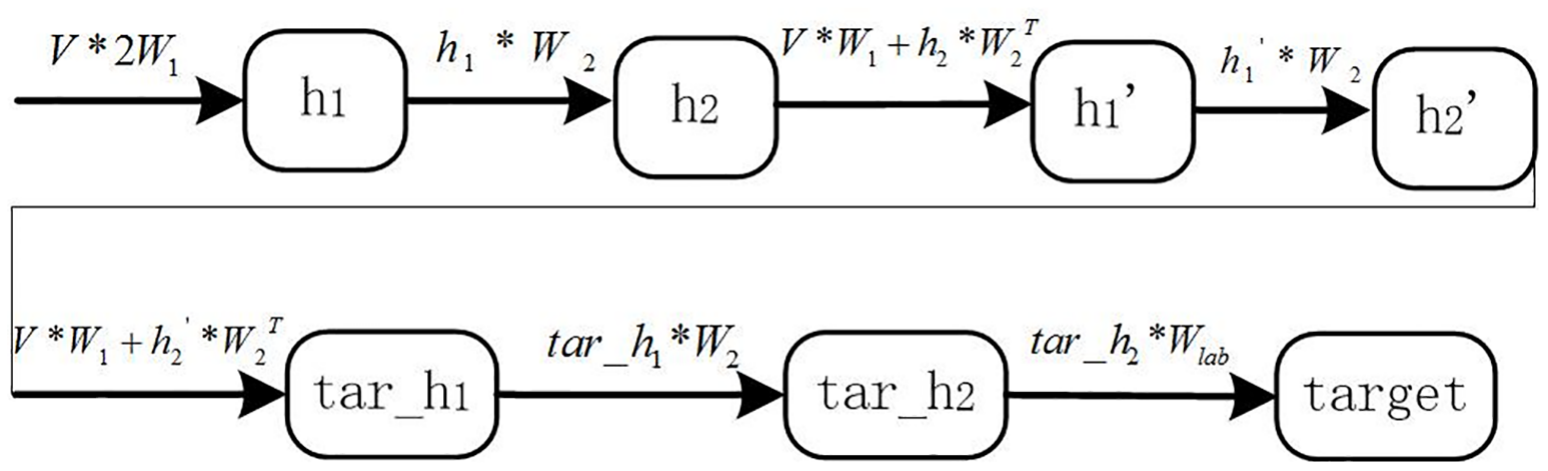
Testing process.

The node values in the ***h***_**1**_ and ***h***_**2**_ hidden layers are calculated according to the nodes in the previous and next layers. After the unsupervised pre-training process, we used a supervised fine-tuning process in which we introduced the labels of the samples. The purpose of the fine-tuning is to update the weights connecting visible (hidden) and hidden layers, and the objective function is to minimize the energy function in the model. The DBM is an undirected graph model, in which each neuron node in the same layer is computed according to the adjacent related lower and upper nodes. As the test sample labels are unknown, unlike the training sample labels, the training and testing processes of the DBM model are different. The details are described in the following section (Fig 4 is the training process and Fig 5 is the testing process)(Note that the 2*W*_1_ represents the 2*W*_1_ parameters illustrated in Fig 2 between layer *V* and layer *h*_1_):

In the training process: During the training process, the model is sampled in the initial state and the nodes are reconstructed for each layer. After sampling the hidden layer nodes in the initial state (***h***_**1**_), which are calculated according to the visible layer nodes (***V***), the subsequent calculation has two steps: calculate the status of the nodes in the second hidden layer (***h***_**2**_) (top row in Fig 4), and reconstruct the status of the nodes in the visible layer ($V^{{}^{\prime }}$) (the lower row in Fig 4). Next, the labels of each sample are calculated, the status of $h_{1}^{{}^{\prime }}$ and $h_{2}^{{}^{\prime }}$ are determined based on the current status (***h***_**2**_), the error is calculated, and then the weights between the node connections are updated.

In the testing process: During the testing process, according to the DBM model node calculation rules (calculating the node status in the current layer according to the upper and lower layers), the weights obtained through the training process cannot be directly used to predict the test sample target. Indeed, the status of the ***h***_**1**_ layer nodes (termed $h_{1}^{{}^{\prime }}$) must be re-sampled after calculating the ***h***_**2**_ layer nodes. We sampled the status of the nodes in $h_{1}^{{}^{\prime }}$ according to the visual layer and the ***h***_**2**_ hidden layer. We then re-calculated the ***h***_**2**_ hidden layer nodes ($h_{2}^{{}^{\prime }}$) and predicted the labels of the test samples (details in Fig 5).

The rectified linear unit (ReLU) can be used to increase the nonlinear properties of the network, as well as the sparsity, without affecting the receptive fields of the convolution layer [56]. The ReLU function, which is also the neuron’s output, is:

$$f\left(x\right)=\{\begin{array}{cc} x, & x\geq 0\\ 0, & x<0 \end{array}$$

The sigmoid function, which is used widely in deep learning with excessive layers, suffers from the vanishing gradient problem; the training process is therefore difficult. Using the ReLU instead of the sigmoid function in a deep network ensures that the network neurons are modestly sparse after training, thus eliminating the issue of vanishing gradients along with the paths of active hidden units [16]. In addition, the ReLU enables faster network training compared with other options and does not require pre-training or advanced optimization strategies. Indeed, the ReLU can achieve performance comparable to a pre-trained model with the sigmoid function.

The recently introduced technique called “dropout” can reduce over-fitting by decreasing the complexity of co-adaptation of data [57]. Neurons are “dropped out” by randomly setting 50% of the nodes in each hidden layer in the network to 0, which means that 50% do not participate in the forward pass or back-propagation processes. In our system, “dropout” is used in all convolutional and fully-connected layers.

In the testing phase, dropout is regarded as a kind of mean network because for each input sample (which may be one sample or a batch of samples), the corresponding network structures are different but share the hidden node’s weight at the same time. Hidden nodes appear randomly in a certain probability when updating weights in the network. One of the advantages of dropout is that it guarantees that every node does not appear at the same time. Hence, weight updating no longer depends on the interaction relationship between each pair of hidden nodes. This can prevent certain characteristics from affected by other specific characteristics so that each individual hidden unit can learn useful features without relying on other specific hidden units to correct its mistakes.

On the one hand, CNN has a great advantage on feature engineering. On the other hand, DBM shows great capability in generating the labels for documents while suffers from large number of parameters, which would lead to unbearable time cost. Compared with them, B-CNN is a combination of CNN and DBM, while the number of hidden layers in DBM can be reduced, replaced by CNN for extracting features from documents. As a result, in terms of time consumption, obviously B-CNN requires a larger computation cost than simple CNN or DBM with two hidden layers due to its complex pipeline. However, these methods cannot achieve the performances of B-CNN. Or if adding hidden layers to DBM, the time consumption cannot be controlled. Actually in practice, the feature engineering process, including CNN and meta feature extraction can be accomplished at the same time. With the optimized parameters, the testing process would not be influenced by the complex architecture that much.

### 3.4 Hierarchical indexing

Document classification aims at predicting the categories of an unknown document in a certain class collection. The task can be categorized into two groups through the number of target classes. Multi-class classification [58] is a common task in which there are obvious boundaries between each pair of classes and a given document can only belong to one class. Binary classification is a classical application of multi-class classification. Another task is multi-label classification [58], where each sample can be assigned with more than one class. The target labels can be either independent or associated which brings more challenging work for classifiers.

As a result, multi-label classifications are not simply repeated multi-class classifications due to the dependencies among labels. In fact, appropriate use of the dependencies can effectively improve the performances of classification. In case there are a large number of labels, through unsupervised clustering as pre-processing, the labels can be grouped into coarse subsets, which can greatly improve the efficiency of classification. Recently, word2vec [59] was developed to represent the word semantics from corpus through a continuous vectors, which has been found useful for obtaining the relationship between words by letting related or compositional words appear closed in the vector space. Based on this toolkit, Ioannis Pavlopoulos trained the word representation for biomedicine labels (http://participants-area.bioasq.org/), which is applied in our research. Based on the word vector representation, we design an indexing structure that introduces label (word) embedding for multi-label classification (shown in the third part of Fig 1). In our framework, the error function of the clustered coarse classes (categories) and the weight updating are used as follows.

$$\begin{array}{c} E=\frac{1}{NC}\sum_{C}\sum_{N}{\left(f\left(x,w\right)-y_{c}\right)}^{2},\;\;\;\;\Delta w=\frac{\partial E}{\partial w}, \end{array}$$ (10)

where *C* is the number of coarse clustering categories, *N* is the number of training samples, *x* is the output label of B-CNN, *w* is weights which connect the output layer and the coarse cluster layer, *f(x, w)* is the output probabilities of a certain label while y is the target value. For each *C*, the hierarchical classification function is:

$$\begin{array}{c} J_{k}=\frac{1}{n_{k}}\sum {\left(f(x,w_{k})-y\right)}^{2},\;\;\;\;\Delta w_{k}=\frac{\partial J_{k}}{\partial w}, \end{array}$$ (11)

where *k* is the number of categories within the corresponding coarse classes subset. The whole process is firstly clustering the labels into coarse classes with word2vec, and then deciding the final label within the coarse classes.

For forward propagation, through Eqs (4), (5) and (6), the output of the whole network can be computed with the input vectors and the parameters of CNN and DBM. Note that *y*_*c*_ can be represented by a function of input vectors and the parameters of CNN. For backward propagation, the parameters in CNN and DBM can be optimized simultaneously through stochastic gradient descent on the cost function.

## 4 Semantic feature representation

Currently, the main feature representation method is the vector space model with the BOW. However, the BOW model only contains information about word frequency, which is shallow and insufficient when dealing with NLP tasks, e.g., biomedicine indexing. In this paper, we describe a novel method for vector representation with document word sequence embedding (DSE) for biomedicine documents, which is based on word sequence information. Our goal was to improve the feature representation (input of the framework). This model enables us to take advantage of the strength of the CNN, which is more suitable for dealing with the features of sequence relationships. We used the sequence of words in the documents as the basic features, and combined categorical information from Wikipedia with entity meta-features constructed from MetaMap keywords from biomedicine fields as a synonym for expansion. Afterwards, we used word embedding to represent these features, rendering this feature representation more informational for biomedicine tasks.

In the following, we first describe the meta-features composed via DSE representation, and then introduce the algorithm in detail. Wikipedia is a multi-language encyclopedia, covering a wide range of information on individual wiki pages. Wikipedia is an actual corpus, and with the rapid changes in social information, it is constantly being expanded and updated. Wu [60] exploited Wikipedia as a source of external knowledge for extending documents. Considering the nature of our task with biomedical abstracts, we adopted this idea and used Wikipedia to extend the documents. Named entity occupies an important position in addressing NLP tasks, e.g., information extraction and information retrieval. Named entity is a representative feature in biomedicine [61], e.g., the prediction of gene sequences and identification of proteins. Word vectors [59] are a good tool for the representation and mining of existing semantic relationships between words. Among existing deep learning methods, many models that obtain good performance are based on word vectors. Thus, for the input of the model, we used word vectors to represent Wikipedia and the named entity features.

### 4.1 Meta-features

#### 4.1.1 Wikipedia as meta-feature enrich document representation

**Fig 6:**
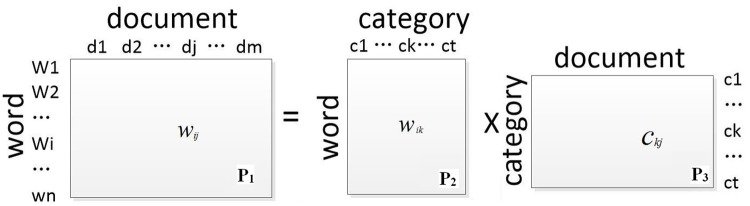
Words mapping with Wikipedia.

For the task of indexing biomedical documents, we enriched the document representation by using Wikipedia category information together with MetaMap keywords. This enabled us to overcome the shortcomings of the BOW. MetaMap (http://metamap.nlm.nih.gov/) is a widely used open source toolkit that extracts concepts in the UMLS metathesaurus. We computed the distribution of categories-words according to the known distribution of word-documents and category-documents using a process referred to as LDA (see Fig 6). This is discussed later in this document.

Parameter description: *n* is the number of distinct words in the documents, *m* is the total number of classes in all documents, and *t* is the number of Wikipedia categories. Matrix **P**_**1**_ represents the distribution of words and documents, where the row ***w***_***i***_ is the probability that the word *w*_*i*_ will appear in *m* classes of documents, denoted as *w*_*ij*_. Matrix **P**_**2**_ represents the distribution of words and Wikipedia categories, where the row ***w***_***i***_ is the probability that a word *w*_*i*_ will correspond to a Wikipedia category, denoted as *w*_*ik*_. Matrix **P**_**3**_ represents the distribution of Wikipedia categories and document classes, where the row ***c***_***k***_ is the probability that the category *c*_*k*_ will appear in the documents, denoted as *c*_*kj*_.

We were able to use the Wikipedia categories for the corresponding anchor words to calculate the Wikipedia categories of non-anchor words (**P**_**2**_). This form of document representation combines global (the proportion of the words in all documents categories) and local (anchor words in each document category) information. Hence, through the text representation, we were able to learn more semantic information via the model.

#### 4.1.2 Entity as meta-feature enriches document representation

Conditional random fields (CRFs) are undirected statistical graphical models that have been used successfully in a large number of studies on named entity recognition. CRFs take advantage of sequence labeling, which is a linear chain that corresponds with conditional training to treat each sentence as a sequence of tokens. In this paper, we used the Machine Learning for Language Toolkit (http://mallet.cs.umass.edu/) (MALLET), as an implementation of CRFs [62], to recognize entity class in biomedicine abstracts. We used biomedicine entities to enrich document representation in two different ways. First, we used the entity properties in the DSE, and second, the entities were included in the DBM inputs as an entity feature vector.

#### 4.1.3 Word vector

The word vector model can be seen as a language model that consists of n-dimensional continuous valued vectors, where each dimension of the word embeddings expresses a latent feature of the words, exposing useful semantic and syntactic regularities [59]. The vector creates features without human intervention. Based on individual words and random initialization, it can make highly accurate guesses about the meaning of each word with enough data, usage, and context. These features are also called the distributed representation of the words.

Mikolov trained word representations using an effective neural network model that seeks to maximize accuracy while minimizing computational complexity [63]. The representation of vectors after training not only positions similar words close to each other in the vector space, but can also help to define implicit relationships among words across a distance. For example, the word vector (“King”) − vector (“Man”) + vector (“Woman”) can have specific properties such that it results in the vector that is closest to the vector representation of the word Queen [64].

### 4.2 Document word sequence embedding (DSE)

We preprocessed all documents by removing stop-words and stemming. To fix the length of all document input features, we generated the following process. The document is represented as follows: if the document length after preprocessing is less than the average length *L*, we use the *Unknown* word as a supplement until the document length reaches *L*. If the document length is longer than *L*, we reprocess the document and retrieve high frequency words until the length of the document matches that of *L*, and then use word embedding according to the sequence of the words in the document. This process was termed algorithm 1.

We sorted all words in a document *k* according to their frequency in the document. We then collected the top words in the document until the document length equaled *L*. ***D*** represents documents after the process of removing stop-words and stemming, ***D***_***k***_ represents document *k*, and *Unknown* refers to the word *Unknown*, which is used for effective word embedding in a large corpus. When the document length is less than *L*, we use this word to supplement the length. *L* − *m* in *Unknown*_*L*−*m*_ refers to the number of *Unknown* words. If the document had only one *Unknown* word, we just used *Unknown*_1_. ***R***_***km***_ refers to the word embedding of the *m* word in document *k*, and ***R***_***newkm***_ describes *m* word embedding in the sequence of new words after the document is rearranged.

**Algorithm 1: Document word sequence embedding (DSE)**

**Input: *D***

**Output:** document representation *R*

1. *L* = average length of *D*

2. for *k* = 1..*N* do

3. *m* = length (*D*_*k*_)

4. if *m* < *L*

5.  *R* = [*R*_*k*1_, *R*_*k*2_, …, *R*_*km*_, *Unknown*_1_, …, *Unknown*_*L*−*m*_];

6. else if *m* = *L*

7.   *R* = [*R*_*k*1_, *R*_*k*2_, *R*_*k*3_, …, *R*_*km*_];

8. else if *m* > *L*

9.   sort all words in document *k* according to their

   frequency in this document. Then take the top words

   in all place of this document until the length equal

   to *L*. *R* = [*R*_*newk*1_, *R*_*newk*2_, …, *R*_*newkm*_, …, *R*_*newkL*_];

10. end if

11. end for

12. Return *R*

In addition, we use the entity and Wikipedia categories for the corresponding anchor words to enrich document representation [65]. These were based on MetaMap keywords in the dataset of the biomedicine literature. Some words in the document may be both biomedical entities and also anchor words in Wikipedia. In these cases, there may be substantial corresponding category information. Taking into account the integrity of the model input, we selected three words to enrich document representation for each word in the ***R***. These words were selected from the corresponding categories and entities. The extension of word order followed that of the original words. Then, word embedding was used to represent each word. When the number of categories was less than three, we used the word itself in the document, together with its categories. Each sentence in the document was thus represented by word embedding, in which each word was represented by one 50-dimensional vector. As a consequence, each document was represented by a 200 (50×4)×L matrix. The details of the process are shown in the feature representation section of Fig 1.

## 5 Experiments

### 5.1 Dataset and experimental setup

Dataset 1 (http://tinyurl.com/m2c8se6) was a labeled corpus of English scientific medical abstracts. All data were downloaded from the Springer website. Dataset 2 (http://biotext.berkeley.edu/data.html) was a collection of BioText data. Dataset 3 (http://www.bioasq.org/participate/challenges) was a group of MEDLINE documents. For dataset 3, we downloaded all data for the years up to and including 2013 from the website. Each article contained only the article title and abstract, and there were 5 to 20 classes (termed Medical Subject Headings in the medical field (MeSH)) for each article on average. Altogether, there were 27149 MeSH headings. Yepes [27] selected the top 10 most frequent MeSH headings to address the extremely unbalanced distribution of the dataset. Similarly, we selected the top 150 MeSH headings because only about 150 of these appear in more than 1% of all the data on MEDLINE. To extend our experiments, we plan to introduce some unbalanced samples and select the top 2000 categories. Dataset 4 (http://qwone.com/~jason/20Newsgroups) was organized into different newsgroups, each corresponding to a different topic. The website offered three versions of this dataset, and we selected the third, which had 18828 documents. This dataset can be used in two ways, as it can be organized into twenty classes or four major categories (comp, politics, rec, and religion). To ensure that our results were comparable to those of Lai [30], we choose to use the same four categories of the dataset 4. For more information about the datasets, see Table 2.

**Table 2:** Datasets details.

Dataset	Sample	Classes	Type	Field
dataset 1	9666	39	multi-class	biomedicine
dataset 2	1000	168	multi-class	
dataset 3	1,000,000	150	multi-label	
		2,000	multi-label	
dataset 4	18,828	20	multi-class	newsgroups
		4	multi-class	

On dataset 1 and 2, we aim to primarily compare the performance of different models and different feature representations. To simplify the complexity, on dataset 3, we choose not to conduct the experiment on the *BOW*_+_ feature representation because of the unsatisfying performance, compared with DSE. Dataset 4 is used to verify the extensions of the proposed model on fields other than biomedicine, so we directly compare our model with state-of-the-art models in the chosen field, rather than the baselines of the former datasets.

These experiments are repeated ten times. In order to avoid insufficient testing set to validate the effectiveness, as well as to guarantee the training set sizes, at each time, 70% of the entire dataset is randomly selected as training samples, and the remaining 30% as testing samples. The presented results are the average of results of the repeated experiments. Moreover, for each, within the selected training samples, 10-fold cross validation is employed to select the optimal parameters. The parameters of the B-CNN model include the settings of the convolutional and pooling layers in CNN, the size of the sliding window in CNN, the interlayer connection weights (e.g. ***W***_**1**_, ***W***_**2**_, and ***W***_***lab***_ in DBM, the connection parameters of weights from the high-level representation layer in B-CNN, and the initial values in the pre-training process and the overall learning rate, and so on. We applied the widely-used cuda-convnet package to train our model on a single GPU. The experimental performance presented below was conducted entirely with the test set.

### 5.2 Details

Based on our DSE feature extension, we also enriched BOW representation through Wikipedia and the entity class based on MetaMap. We used this as our baseline, denoted as *BOW*_+_ (*BOW*_+_ is better than BOW representation. *BOW*_+_ adopts indeed the same representation approaches of conventional BOW). In addition, we made some changes when training CNNs. As our purpose was to learn about the structure of the document and the semantic information between words, we fixed the size of the slide window to make it equal in dimension to that of word embedding (50-dimension). We used a step down sliding window position of 50 by 50, as this prevented each sliding window from changing the word vector embedding associated with the features of the word. In this way, information about the document was gathered by changing the length of sliding window.

#### 5.2.1 Evaluation protocols

We used Macro- and Micro-averages of the precision (P), recall (R), similarity (S), and *F*_1_-measures as the evaluation criteria [66–69]. The Macro-average weights all the classes equally, regardless of how many documents are included. The Micro-average weights all the documents equally, thus favoring the performance of common classes. Hence, the Macro-average reflects the performance of each category, and the Micro-average reflects the performance of each document. MacroP (MaP), MacroR (MaR), Macro*F*_1_ (Ma*F*_1_), MicroP (MiP), MicroR (MiR), and Micro*F*_1_ (Mi*F*_1_) are comprehensive assessment metrics, defined as:

$$\begin{array}{c} MacroP=\frac{\sum_{k=1}^{K}P_{k}}{K}, \end{array}$$ (12)

$$\begin{array}{c} MacroR=\frac{\sum_{k=1}^{K}R_{k}}{K}, \end{array}$$ (13)

$$\begin{array}{c} MacroF_{1}=\frac{\sum_{k=1}^{K}F_{1k}}{K}, \end{array}$$ (14)

$$\begin{array}{c} MicroP=\frac{\sum_{k=1}^{K}TP_{k}}{\sum_{k=1}^{K}TP_{k}+\sum_{k=1}^{K}FP_{k}}, \end{array}$$ (15)

$$\begin{array}{c} MicroR=\frac{\sum_{k=1}^{K}TP_{k}}{\sum_{k=1}^{K}TP_{k}+\sum_{k=1}^{K}FN_{k}}, \end{array}$$ (16)

$$\begin{array}{c} MicroF_{1}=\frac{MicroP\times MicroR\times 2}{MicroP+MicroR}, \end{array}$$ (17)

where *K* is the number of categories. Precision (P) is defined as: $P=\frac{TP}{\left(TP+FP\right)}$ and recall (R) as: $R=\frac{TP}{TP+FN}$. *F*_1_, Macro-similarity (MaS) and Micro-similarity (MiS) are defined as:

$$\begin{array}{c} F_{1}=\frac{2\times P\times R}{\left(P+R\right)}, \end{array}$$ (18)

$$\begin{array}{c} MaS=MiS=\frac{TP}{\left(TP+FP+FN\right)}, \end{array}$$ (19)

where *TP* refers to the number of true positives, *FP* is the number of false positives, *FN* is the number of false negatives, and we also use *TN* which is the number of true negatives in the Roc graph.

A ROC [70] graph depicts the relative tradeoffs between the benefits (*TP*, true positives) and costs (*FP*, false positives). ROC curves are two-dimensional graphs in which the TPR (true positive rate) is plotted on the Y axis and the FPR (false positive rate) is plotted on the X axis. In the ROC curve, the closer the point to the top left, the better the model. The TPR and the FPR of a classifier are estimated as:

$$\begin{array}{c} TPR=\frac{Positive\;correctly\;classified}{Total\;positives}=\frac{TP}{\left(TP+FN\right)}, \end{array}$$ (20)

$$\begin{array}{c} FPR=\frac{Negatives\;incorrectly\;classified}{Total\;negatives}=\frac{FP}{\left(FP+TN\right)}. \end{array}$$ (21)

#### 5.2.2 Comparative methods

We compared the performance of our best model (with five pooling layers, each of which follows a convolutional layer, and three fully-connected layers at the end of CNNs) with MTI, MeSHUP, which we have described in our related work, and typical methods, as follows.

**Pattern Matching (PM)** [19]: By comparing MeSH labels with words in the documents, we were able to predict document labels. During the matching process, we added some artificial rules: convert all words in the MeSH label into lowercase. In addition, we determined the characters in MeSH labels using fuzzy matching. For example, it might be unlikely to see *pain* *bone* in a MeSH label, but very likely to find *pain* *in* *bone* in a document.

**Latent Dirichlet Allocation (LDA)** [33]: The LDA is a directed graphical model, also referred to as the topic model. This model is an unsupervised learning technique for extracting thematic information from a corpus. LDA, HLDA (Hierarchical LDA), and PLSA (probabilistic latent semantic analysis) have been extensively used for classification because the nonparametric extensions of these models have been quite effective [71]. The main purpose of LDA is to reduce the dimensions of document representation using thematic dimension representations instead of the dictionary. This motivation of the LDA method is similar to that of deep learning, in which features are extracted from high-dimensional space. Variational EM algorithms and Gibbs sampling are used in the LDA model, which makes two of the parameters, i.e., document-topics and topic-words, easier to predict.

**Support Vector Machines (SVM)** [72]: In machine learning and data mining, the SVM is supervised learning models with associated learning algorithms that analyze data used for classification and regression analysis. In this paper, the multi-label classification produced by SVMs is regarded as a group of multiple binary classification problems. The samples from each class in the dataset have an unbalanced distribution. For each class, positive samples are defined as current class samples, and negative samples are two to three times the size of the positive samples. As described in the reference [73], when the number of features is small, one often maps data to higher dimensional spaces (i.e., using nonlinear kernels). Considering the datasets in the experiments, the RBF (Radial Basis Function) kernel is selected and used which has two parameters, C and *γ*. In SVM, the RBF kernel is selected by considering two factors: the first reason is that this kernel nonlinearly maps samples into a higher dimensional space; correspondingly the second reason is the number of hyper-parameters that influences the complexity of our model. In the experiments, cross-validation was applied to select the best pair of (C, *γ*) from various pairs of candidate parameters. In addition, we also tried the linear kernel (LIBNEAR) to compare with LIBSVM with the RBF kernel.

**Naive Bayesian (NB)** [74]: The NB is a supervised probabilistic learning method. The basic idea of this method involves the calculation of appearance probability under some class, returning the label of the maximum probability.

**Logistic Regression (LR)** [74]: The most simple form of regression is linear regression. In LR, a logic function is applied on the basis of linear regression. In LR, a set of weights is learned by training with classifiers. In the test phase, the set of weights can discriminate results according to training samples. Unlike LR, which uses logistical loss as the objective function, the SVM uses the hinge loss function. The SVM method considers support vectors, which are the most relevant points for defining the classification boundary when training the classifiers. Alternatively, the LR method uses nonlinear regression mapping, which greatly reduces the weight of points that are far away from the classification boundary, and relatively improves the weight of the most relevant points.

**Hierarchical CNN (HC)**: In HC, the feature representation of a document is learned by CNN training only, and then hierarchical classification is conducted.

**CNN+DBN**: We verified the validity and necessity of the DBM component of our B-CNN model by using DBN instead of DBM for biomedicine indexing. DBN is also a good feature extraction method in deep learning, and like DBM, is composed of stacked RBMs. However, the two differ in that the DBN is a directed graph model, that calculates the hidden layer nodes based on the previous layer nodes only, while the DBM is an undirected graph model, in which the state of hidden layer nodes is decided jointly by the upper and lower layers (the detailed calculation process is shown in Figs 4 and 5). To compare the DBM and DBN in this paper, we selected two-layer RBMs for feature representation.

**Directly Binary Classification (DBC)**: In our experiments, in order to verify the validity of the hierarchical classification in our model, we compared our method to flat classification based on the CNN model, called DBC. For each class, a binary classification can be utilized to extend each node into two nodes. The labels are modified for each document as follows: if they are labeled (1, 0), it means the document belongs to this class, and if they are labeled (0, 1), it does not belong to the class. The output values of the nodes are computed by the sigmoid function. Comparing the values of the two nodes determines whether the document belongs to a specific class.

### 5.3 Experimental results

In this section, we analyzed the model performance using 11 models with four evaluation metrics, four datasets, and two feature representations. We conducted experiments in three parts. In the first part, we compared and analyzed biomedicine indexing performance with different feature representations using dataset 1 and dataset 2. In the second part, we analyzed the effects of the different models in terms of features representations using dataset 3. ROC analysis and significance tests are described in the third part.

We present the experimental results for the 11 methods based on the DSE and *BOW*_+_ features in the tables listed in the experimental results section for dataset 1 and dataset 2 (horizontal comparative analysis of the models with different features, comparative analysis of the longitudinal effects of different models with the same features). The figures show the experimental results for dataset 3. As dataset 4 was not from a biomedical field (we wanted to verify the extensions of the B-CNN model), these data are analyzed separately in section 5.4.

#### 5.3.1 Experiment with different feature representations

When comparing *BOW*_+_ in terms of DSE features, each document is represented as a matrix based on the DSE feature or as a vector based on the *BOW*_+_ feature. The SVM, LDA, LR, and NB methods involve learning global features, so the input features (DSE) of the document are read by the columns of the matrix during training; that is, each document is represented as a vector of 200×*L*.

As can be seen in Tables 3, 4 and 5, our B-CNN method with DSE feature representation had the best performance among the models. However, for the shallow learning (except HC, CNN+DBN, and DBC) models, the DSE was not superior to the *BOW*_+_. This is likely because these models learn using global features, and different documents in the same dimension can be represented by the same word in BOW feature representation. Conversely, the DSE cannot distinguish between different words in these shallow learning models, and so there is an increase in noise.

**Table 3:** Classification results (%) on dataset 1.

Method	*BOW*_+_						DSE					
	MiP	MiR	Mi*F*_1_	MaP	MaR	Ma*F*_1_	MiP	MiR	Mi*F*_1_	MaP	MaR	Ma*F*_1_
**PM**	21.90	31.56	25.86	22.12	39.45	28.35	21.90	31.56	25.86	22.12	39.45	28.35
**LDA**	55.15	54.77	54.96	56.28	55.89	56.08	50.98	50.98	50.98	51.49	50.98	51.23
**SVM**	57.44	57.04	57.24	58.02	57.61	57.82	47.43	47.00	47.21	47.05	46.63	46.84
**NB**	53.47	55.86	54.64	55.70	55.31	55.50	50.53	49.93	50.23	50.64	50.43	50.53
**LR**	54.98	53.54	54.25	54.54	55.19	54.86	50.56	46.52	48.46	50.97	46.89	48.85
**MTI**	62.03	**63.86**	62.93	62.66	62.61	62.63	52.27	52.11	52.19	52.22	52.06	52.14
**MeshUP**	63.95	59.62	61.71	62.69	62.10	62.40	50.66	50.20	50.43	50.56	50.10	50.33
**HC**	60.65	58.36	59.48	61.89	59.55	60.70	63.20	61.05	62.11	66.93	63.75	65.30
**CNN+DBN**	63.56	61.93	62.73	63.00	61.80	62.40	65.98	64.36	65.16	68.19	64.94	66.52
**DBC**	54.84	52.39	53.59	55.12	52.91	53.99	58.96	57.56	58.25	59.08	58.26	58.67
**B-CNN**	**64.96**	63.30	**64.12**	**64.54**	**62.87**	**63.69**	**68.51**	**66.89**	**67.69**	**70.31**	**66.20**	**68.19**

**Table 4:** Classification results (%) on dataset 2.

Method	*BOW*_+_						DSE					
	MiP	MiR	Mi*F*_1_	MaP	MaR	Ma*F*_1_	MiP	MiR	Mi*F*_1_	MaP	MaR	Ma*F*_1_
**PM**	25.57	36.78	30.17	25.27	36.78	30.17	25.57	36.78	30.17	25.27	36.78	30.17
**LDA**	47.27	46.94	47.11	47.04	46.00	46.52	40.06	39.66	39.86	40.10	39.70	39.90
**SVM**	48.74	49.71	49.22	48.79	49.76	49.27	40.96	40.59	40.77	40.87	40.14	40.50
**NB**	46.79	46.46	46.62	45.71	44.97	45.34	40.68	40.28	40.48	39.46	38.67	39.06
**LR**	45.81	45.90	45.86	45.03	45.12	45.08	38.11	35.06	36.63	38.30	36.05	37.14
**MTI**	52.01	51.96	51.99	49.93	50.92	50.42	42.28	42.16	42.22	44.52	41.31	42.86
**MeshUP**	51.41	52.90	52.14	51.51	**53.06**	52.27	39.54	39.19	39.37	39.47	40.09	39.78
**HC**	52.00	50.51	51.25	51.39	47.32	49.27	56.01	54.23	55.11	53.21	50.43	51.78
**CNN+DBN**	54.70	52.57	53.61	53.85	50.07	51.89	58.60	56.19	57.37	55.87	52.50	54.13
**DBC**	47.30	43.51	45.32	46.96	43.08	44.94	49.50	50.50	49.99	48.95	48.93	48.94
**B-CNN**	**56.52**	**54.46**	**55.47**	**55.97**	52.51	**54.18**	**60.94**	**58.49**	**59.69**	**58.92**	**55.94**	**57.39**

**Table 5:** Similarity measure (%) on dataset 1 and dataset 2.

Method	dataset 1				dataset 2			
	*BOW*_+_		DSE		*BOW*_+_		DSE	
	MiS	Mas	MiS	MaS	MiS	MaS	MiS	MaS
**PM**	40.56	41.94	40.56	41.94	42.08	42.08	42.08	42.08
**LDA**	52.61	53.24	50.49	50.62	48.60	48.32	45.40	45.41
**SVM**	53.90	54.24	48.64	48.47	49.62	49.64	45.78	45.66
**NB**	52.45	52.91	50.12	50.27	48.37	47.77	45.65	45.07
**LR**	52.22	52.56	49.28	49.47	48.01	47.65	44.09	44.32
**MTI**	57.44	57.23	51.12	51.09	51.01	50.21	46.39	46.69
**MeshUP**	56.68	57.08	50.22	50.17	51.10	51.17	45.19	45.36
**HC**	55.25	56.00	56.90	59.06	50.64	49.68	52.70	50.93
**CNN+DBN**	57.30	57.08	58.94	59.93	51.89	51.00	53.99	52.18
**DBC**	51.87	52.09	54.50	54.75	47.80	47.63	50.00	49.48
**B-CNN**	**58.23**	**57.94**	**60.75**	**61.17**	**52.90**	**52.21**	**55.38**	**54.02**

While the DSE is not suitable for these shallow learning models, it does show good performance in the CNN model. There are several reasons for this. First, this representation considers the words sequence of the document. As mentioned above, CNNs are more suitable for dealing with features with a sequence relationship, which is also the reason behind their contribution to speech research [14, 51]. Although the words in the same dimension are not fixed, each word has a 50-dimensional word embedding representation that is easily distinguished from that of other words. Indeed, setting the size of the sliding window and learning the local information between words can suppress the emergence of noise. The noise comes mainly from the stochastic gradient descent procedure. It can be attributed to the approximate sampling procedure what cause systematically biased estimates of the gradient [75]. DBM can be trained in a semi-supervised manner with labels connected to the top layer. It can improve performance and simplify the training of deep generative models. It also controls noise and enables training via backpropagation like standard deep supervised networks [76]. Finally, CNNs are trained by convolution, pooling, and ReLU, and these nonlinear layers also represent a good solution for the information interference problem introduced by using *Unknown* word when the document length is less than *L*.

Second, the *BOW*_+_ representation is too sparse, especially in the case of unbalanced data, where the difference between the feature representations of documents is small. Categories with a large number of samples greatly interfere with categories with fewer samples, leading to predictions that are generally biased to high frequency labels.

Summarily, a comparison of our experimental results for DSE and *BOW*_+_ features shows that the DSE (based on word embedding) clearly retrieves more semantic knowledge and extracts a more accurate representation of the document.

#### 5.3.2 Experiment with different models

The HC model, in which the entity characteristics do not enrich the document, has high precision but low recall. Upon further examination of the labels of the classification results, we found that most of the missing labels classified by CNNs are MeSH terms. Few samples belong to these tags in the datasets, so it is not easy for CNNs to capture these features. However, the advantages gained by combining the CNN with the entity features retrieved by the DBM compensate for the tendency of the CNN to focus on local information.

**Fig 7:**
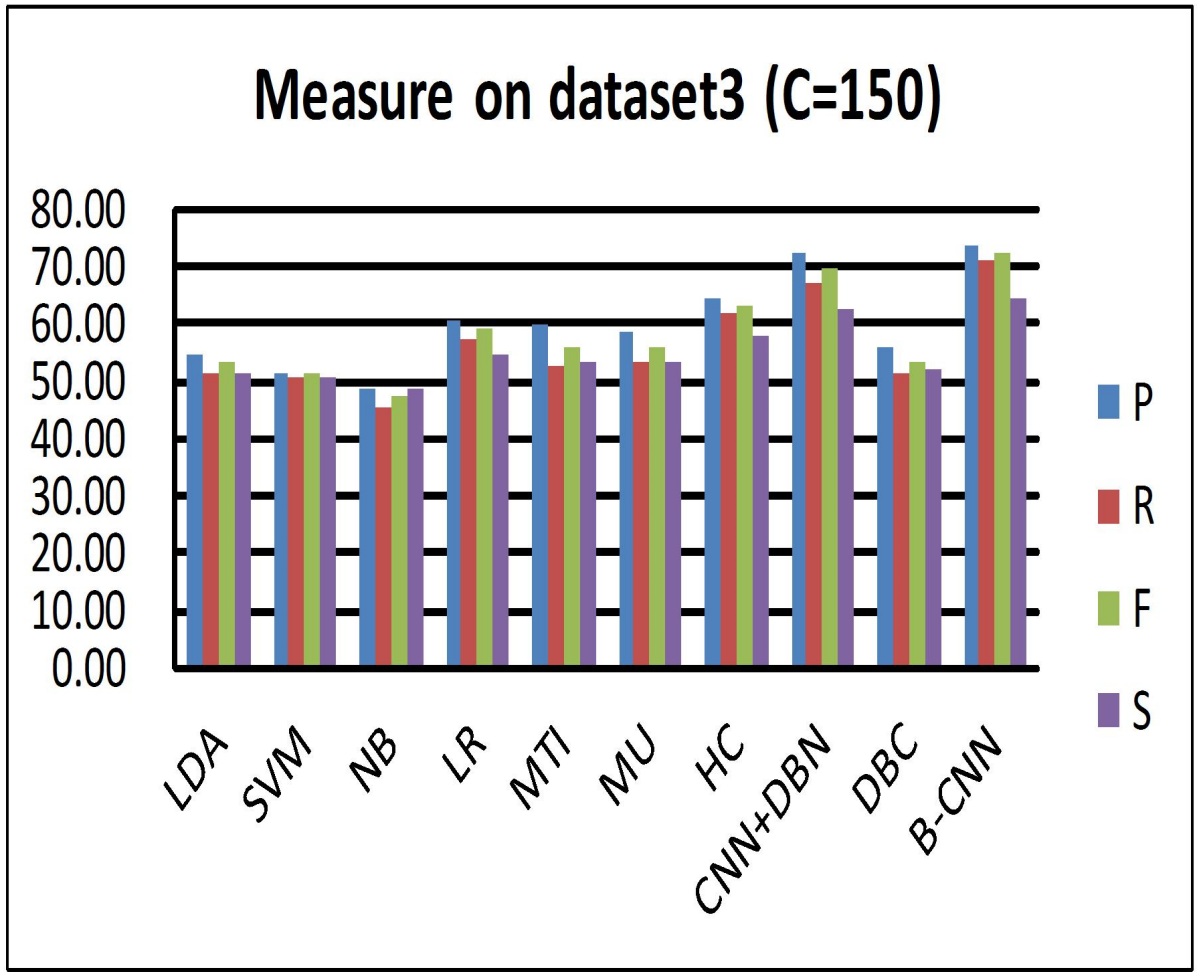
Measures (Precision recall *F*_1_ similarity) on dataset 3 (C = 150).

**Fig 8:**
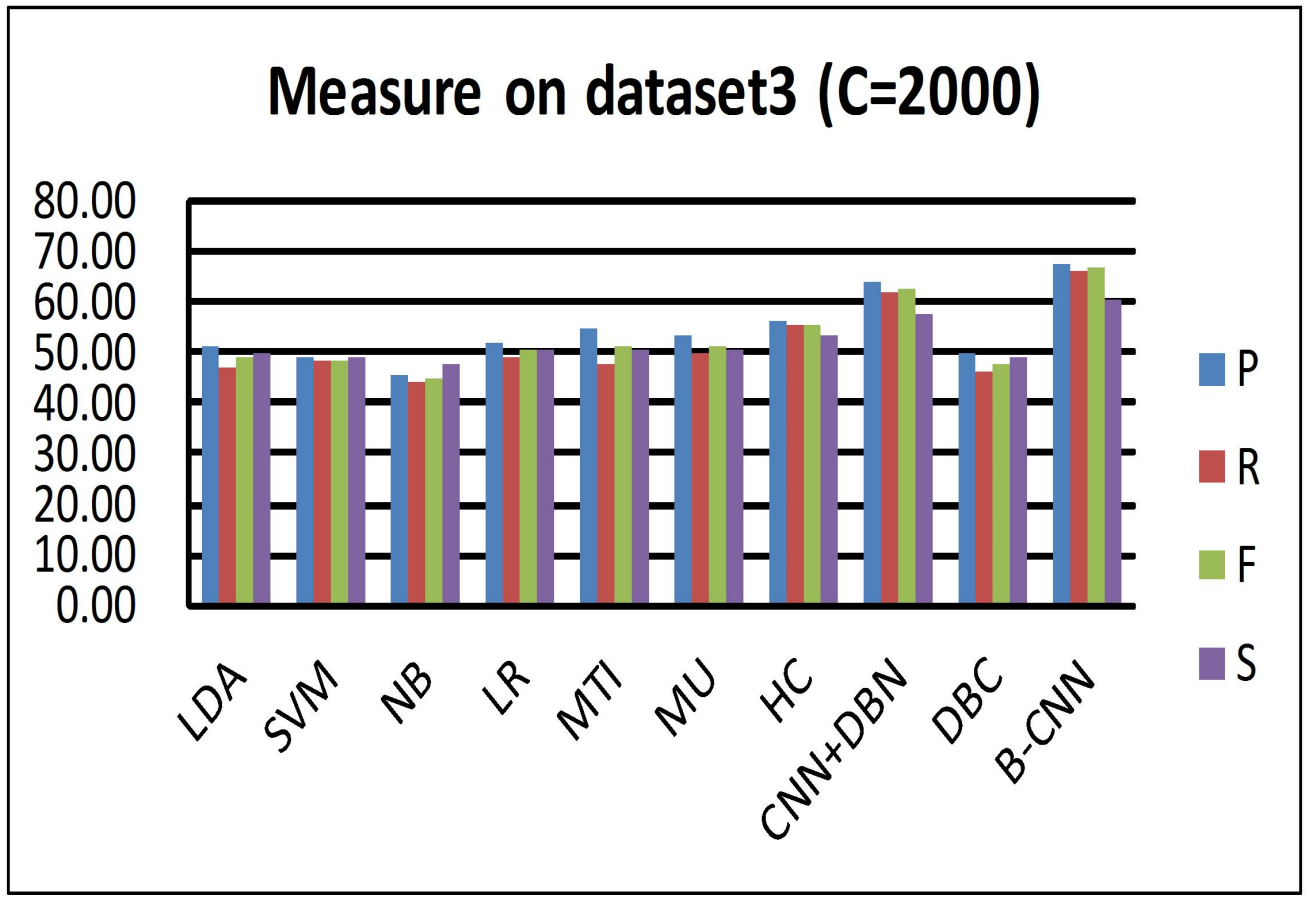
Measures (Precision recall *F*_1_ similarity) on dataset 3 (C = 2000).

**Fig 9:**
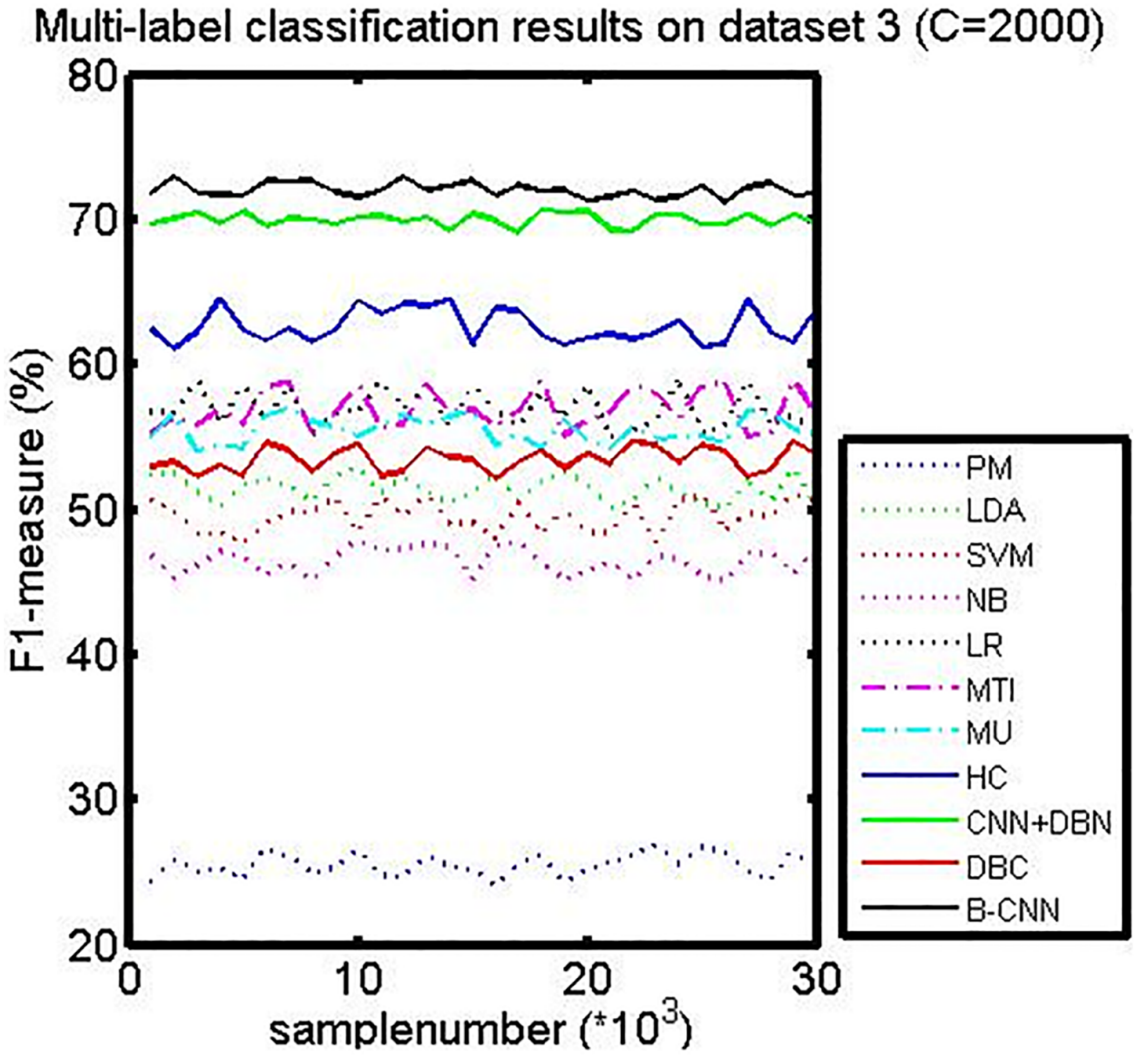
*F*_1_-measure curves on dataset 3.

We compared the CNN+DBN model with a version of the B-CNN model in which the DBM had been replaced by the DBN. From Figs 7, 8, and 9, we can see that the CNN+DBN model is better than the HC model but still not as good as the B-CNN model. This undirected graph model can adjust the weights that connect the nodes between visible and hidden neurons, and it is thus more flexible. To further explore why the B-CNN performs better than the CNN+DBN model, we checked the document representation in the high-level components of the DBM and DBN. We found that the cosine value calculated in the DBN model was larger than that in the B-CNN model. Generally, a larger cosine value indicates that the two samples are more similar. Although the input vector of the document was the same, the representation of the document differed between the two models. The smaller cosine value for the B-CNN model indicates that the test document representation of the model output node dimensions was more refined. This also means that the B-CNN model can learn more deeply distributed representations from documents between different samples, which can help the new test document retrieve more similar samples from the training samples.

For the DBC model, Tables 3 and 4 and Fig 9 (this figure shows the performance of hierarchical classification on the C = 2000 dataset, which has a similar performance on C = 150) all show that hierarchical indexing performed much better than flat classification (DBC) when processing a large number of classes. Moreover, the coarse cluster step is an effective way to remove noise from the unbalanced distributed samples. By analyzing the results from the test documents, we found that most test samples were predicted to be negative by the model. Further evaluation of the training stage revealed very few class numbers of positive samples. Too many nodes were connected in the model, and the adjustment of their weights was not updated in the fine-tuning process. Specifically, more weights were updated in the negative samples compared with the positive samples.

**Fig 10:**
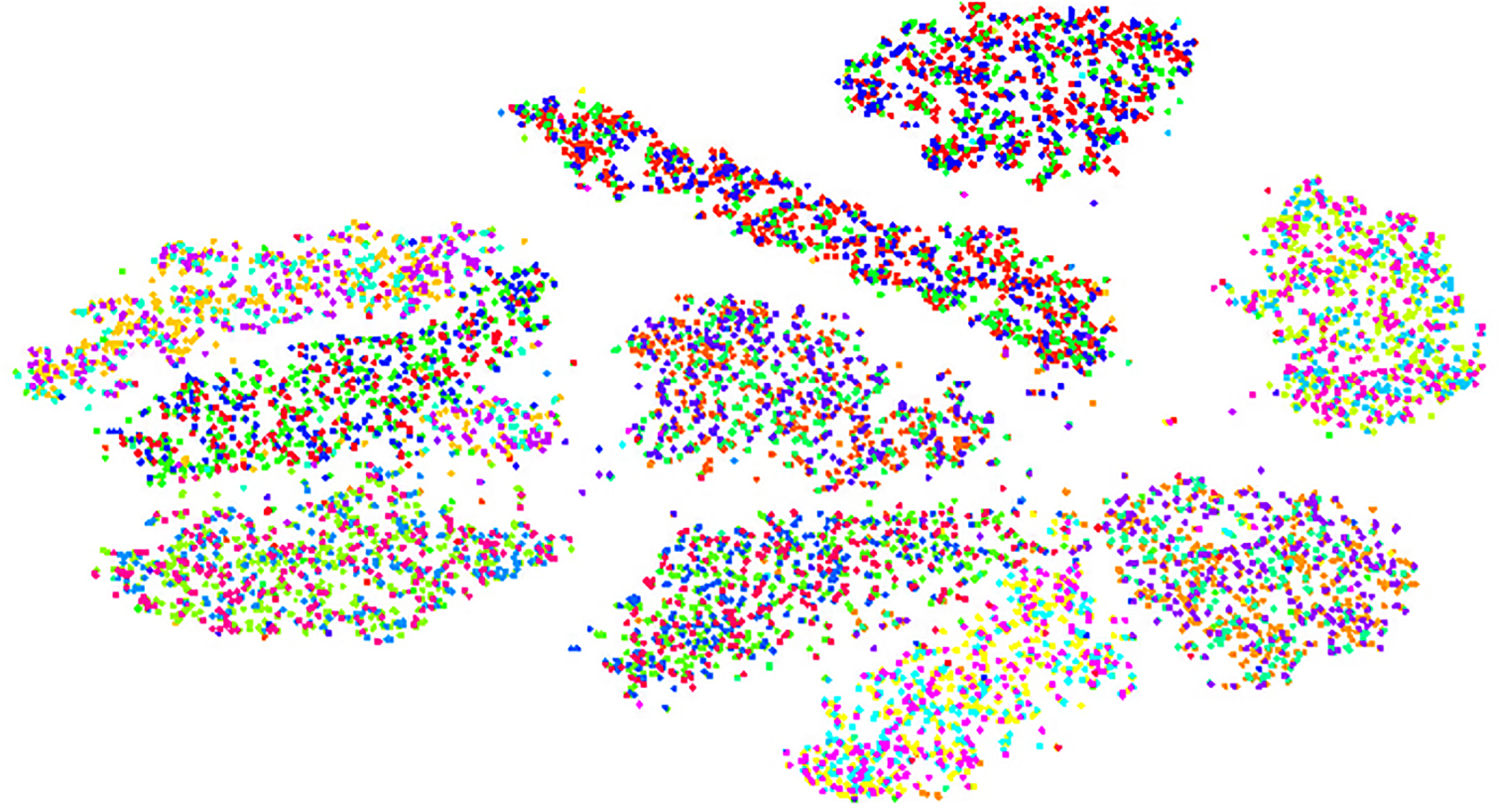
Coarse clusters of label embedding.

Our proposed hierarchical semantic indexing method (B-CNN) greatly increased the classification precision for the positive samples in the first layer. Fig 10 is the label embedding representation of coarse clusters. This figure shows the classification results of the samples, which were previously colored based on the actual classes of the chosen samples. The distances between each two clusters proves the effectiveness of clustering of the proposed model. As shown in the figure, through the coarse classification process, the samples are grouped into 10 obvious clusters. The colors of the samples are based on the co-occurrence of the words. That is, if two labels contain some similar words, they may be in the same or similar colors. The phenomenon whereby the points with the same color are not grouped together indicates the fact that the co-occurrence of the words cannot completely represent the semantics. In summary, the distance between each pair of clusters proves the effectiveness of coarse classification, while the distribution of the same colors indicates that the relations among labels cannot be represented by co-occurrence. Hence, our future research work should consider other relations among the labels to better construct a hierarchical classification tree. This hierarchical semantic framework can effectively reduce the negative impact of negative samples, and mainly updates the weights connected with previous layer nodes during the next layer classification. The updates to weights of nodes that are connected with different previous layer nodes are independent from one other (see Eq 11), which greatly improves the efficacy of this model for training of positive samples. We also compared the ReLU with the sigmoid function in our B-CNN model. The network neurons with ReLU were reasonably sparse after training.

To summarize, the efficacy of our approach owes not only to the hierarchical indexing architecture, but also the feature representation. MetaMap can extract concepts that appear in the UMLS from biomedical texts. These vocabularies can provide better representations for retrieving relevant MEDLINE citations.

#### 5.3.3 ROC analysis

**Fig 11:**
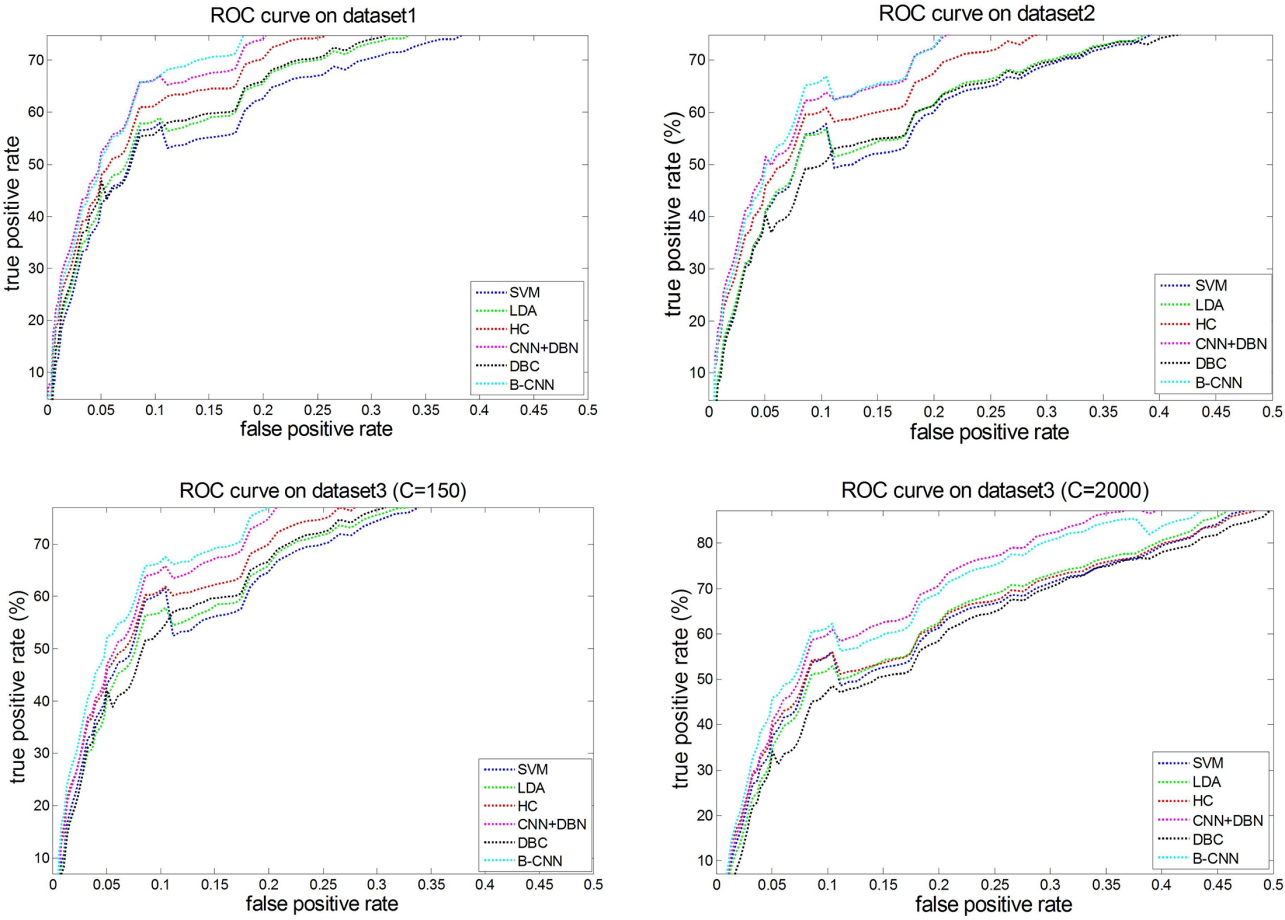
Roc curves on all datasets.

The four ROC curves (shown in Fig 11) for the three biomedical datasets were produced by six different methods (the SVM and LDA are state-of-the-art classification methods in shallow learning, and the others are deep learning methods). The reasons to select the ROC measure to analyze the performances are mainly derived from the effectiveness (compared with precision, recall, *F*_1_-measure, and similarity) and the robustness of different models. These two performances can be effectively evaluated through the trend of the ROC curves of different models. Obviously, the best curve trend is continuously rising. To better analyze and compare the performance of different methods, we amplified and focused on the results of the ROC partial curve (the abscissa range only shows the data values between 0 and 0.5). We followed the papers [77, 78] and downloaded the codes from (http://www.mathworks.com/matlabcentral/profile/authors/1336198-stefan-schroedl).

Fig 11 shows the ROC graphs, from which the performances of different models can be apparently distinguished. In these figures, the closer to the upper left the curve is, the better the performance of the corresponding model is. These curves shows our analysis of the effects of different models on the datasets: (1) all of the curves in the figure are accompanied by a slight fluctuation. We analyze the main reason for the decrease. The major reason is also derived from the robustness analysis of these models. When FPR reaches a certain threshold (about *fpr* = 0.1), the TPR performance decreases, and this decrease is significant in dataset 2. We checked the labels of the samples predicted by these models to examine why FPR increased while TPR performance decreased. It appears that the samples with fewer class numbers (sparse category) were not correctly judged. As there were few samples in these categories, the samples could be overlooked when training the classifiers with these models. Thus, when these categories appeared in the test set, the classifiers lost their identification capabilities, leading to downward curve fluctuations. (2) Fig 11 shows that the effects of our model (B-CNN) on dataset 1, dataset 2, and dataset 3 (C = 150) are superior to those produced by other methods (the curve is the closest to the top left corner). However, for dataset 3 (C = 2,000), the B-CNN model outperforms CNN+DBN only when the FPR is less than 0.1. Otherwise, our model performs more poorly than CNN+DBN. We examined these results by checking the predicted label samples and found that under the same feature space, the document representations learned by the CNN+DBN model were more similar within documents (larger cosine). Thus, when the FPR is low, which also means the error rate requirements are small, the B-CNN has better performance. When FPR increases, the error rate is able to be larger. As the document representation learned by the CNN+DBN model has small differences, making it easier to categorize the documents properly, it is better than the B-CNN model when the FPR is high.

Although our model showed good performance for multi-class and multi-label tasks, and was able to overcome problems caused by unbalanced samples within a certain dimension range, the ROC curve was not always optimal when the number of categories in the unbalanced samples was extremely large (e.g., only 150 of the classes in dataset 3 exceeded 1%). Therefore, we plan to introduce one-shot learning to forecast the characteristics of unbalanced samples in our future research.

#### 5.3.4 Significance test

**Fig 12:**
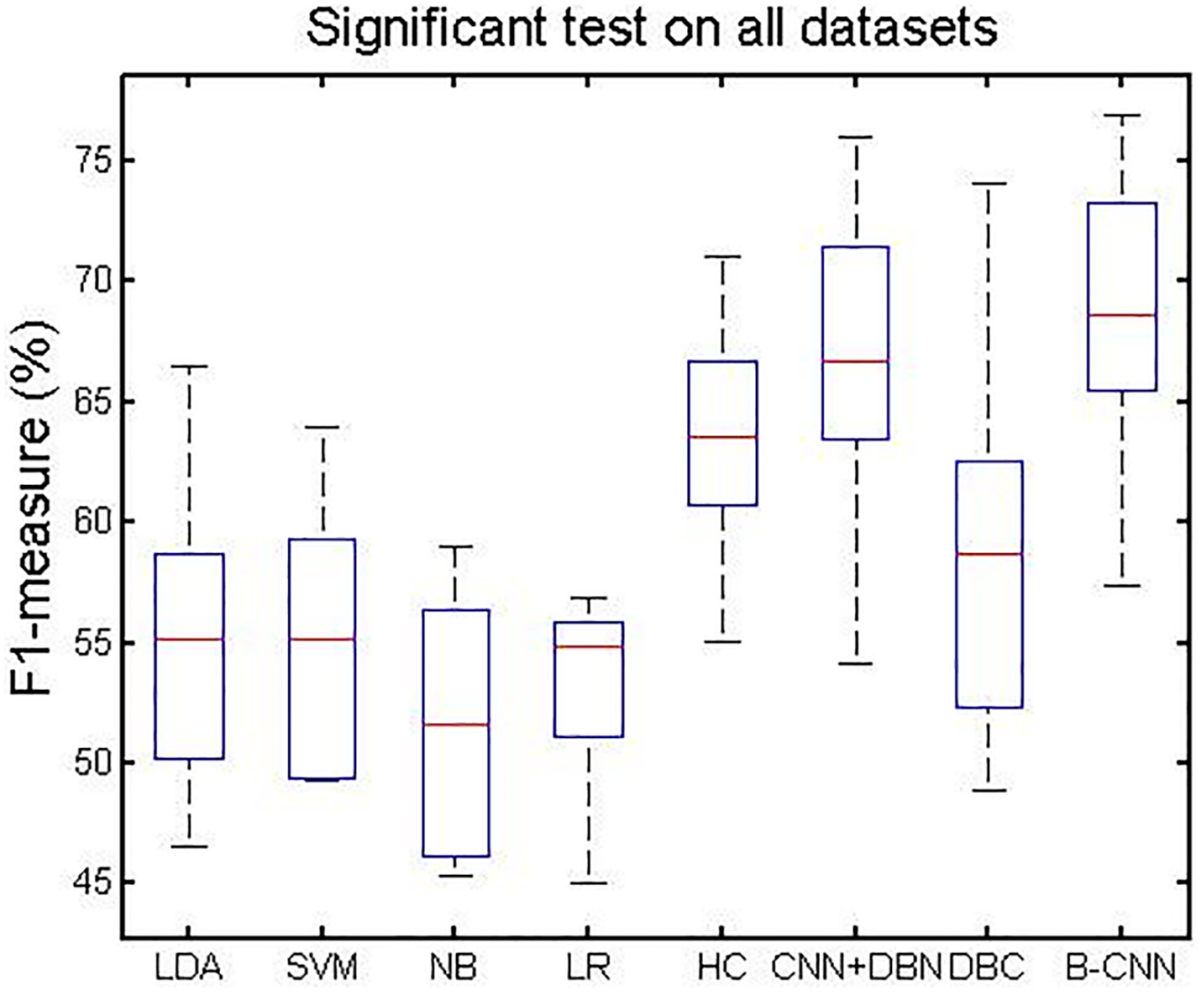
Significant test on all datasets.

Fig 12 shows the *F*_1_-measure comparison of performance between our method and other shallow and deep learning approaches. From this figure, we can observe the following. (1) The deep learning methods had better performance than the shallow learning methods. (2) The hierarchical indexing framework was better than the flat learning methods. (3) Our B-CNN method and the CNN+DBN were ranked first and second, respectively, among all of the methods. This indicates that the feature fusion method between global and local entities in each document benefits from the advantages of DBM.

#### 5.3.5 Friedman test

The Friedman test is a non-parametric statistical test and is used to detect differences in treatments across multiple test attempts. The statistic test is calculated by converting the original results to rank results (the best performing algorithm ranks the 1st, the second best one ranks the 2nd, etc.,). Then, the Friedman test needs the computation of the average ranking [79]. Table 6 shows a comparison of performances with the metric *F*_1_-measure among the eight algorithms selected in the experimental study. With the eight algorithms and the four data sets, *F*_*F*_ is distributed according to the *F* distribution with 8-1 = 7 and (8-1)*(4-1) = 21 degrees of freedom. According to the *F*(7,21) distribution, the computed p-value is lower than 0.01, so the null hypothesis is rejected at a high level of significance. From this table, we can observe the following statements. (1) The proposed B-CNN model achieves the best performance (which is ranked the 1st). (2) The DBC and CNN+DBN models performs slightly poorer than the B-CNN model, and the performance of the CNN+DBN model is superior to that of the DBC because the CNN+DBN is based on the hierarchical classification, which can improve the results greatly.

**Table 6:** Comparison of F measure (%) among the eight algorithms selected in the experimental study. The ranks are used in the computation of the Friedman test.

Data set	LDA	SVM	NB	LR	HC	CNN+DBN	DBC	B-CNN
**dataset 1**	46.52 (6)	49.27 (4)	45.34 (7)	45.08 (8)	55.11 (2)	54.13 (3)	48.94 (5)	57.39 (1)
**dataset 2**	51.43 (6)	49.50 (7)	46.44 (8)	54.86 (4)	62.65 (3)	66.52 (2)	53.45 (5)	68.19 (1)
**dataset 3 (C = 150)**	56.08 (6)	57.82 (5)	55.50 (8)	55.54 (7)	65.30 (3)	69.97 (2)	58.67 (4)	72.02 (1)
**dataset 3 (C = 2000)**	66.48 (5)	63.97 (6)	59.01 (7)	56.89 (8)	71.04 (4)	76.03 (2)	74.03 (3)	76.95 (1)
**average rank**	5.75	5.5	6	6.75	5	3	3.5	1

### 5.4 From biomedicine indexing to newsgroup classification

**Fig 13:**
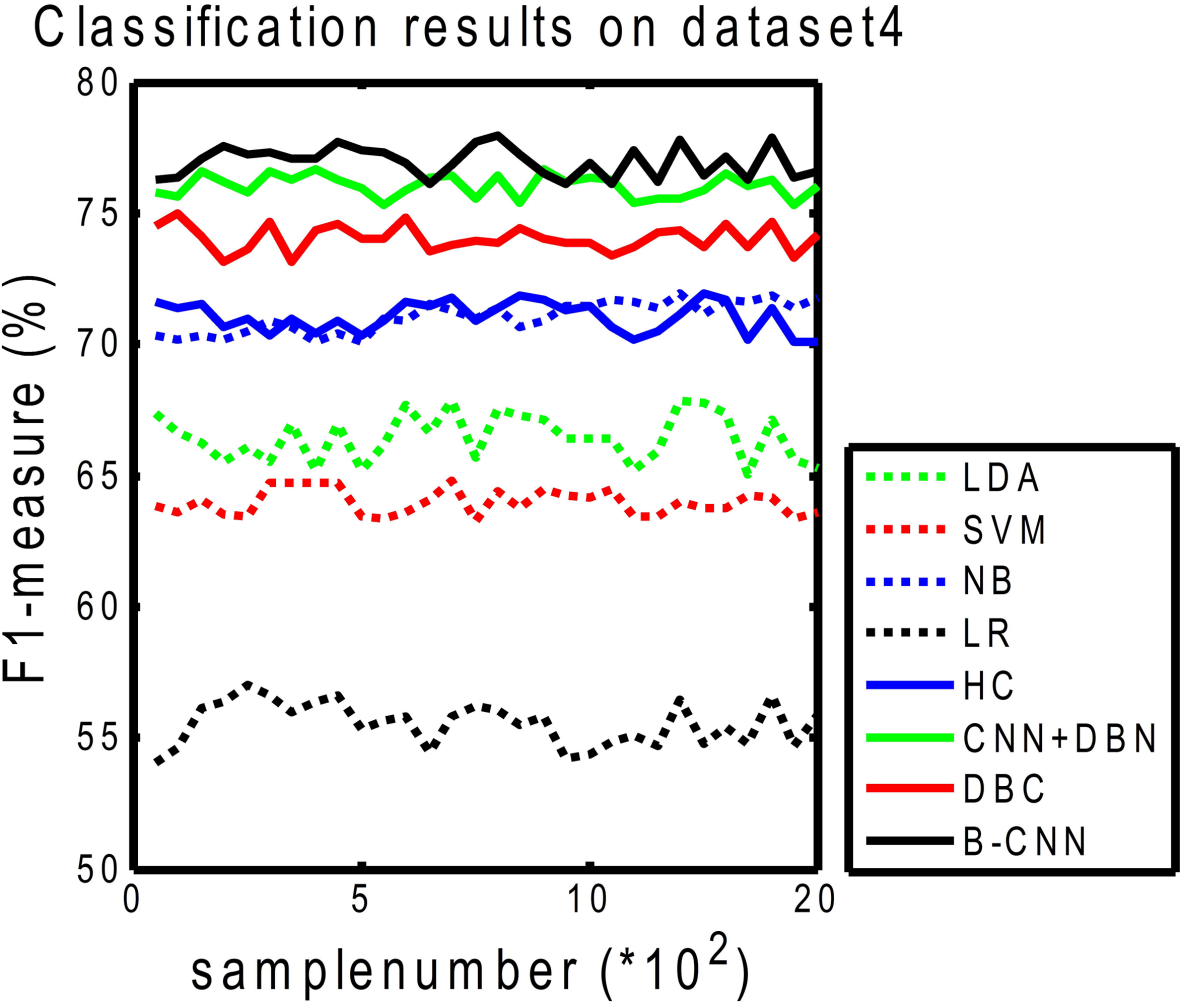
*F*_1_-measure curves on dataset 4 (C = 20).

In Fig 13, we can see that our B-CNN framework achieved good results not only for biomedical indexing but also for other areas of text classification. For dataset 4 (20 newsgroups, C = 20), we choose the LDA extension instead of the entity feature extension, as LDA can be used to represent conventional topics. Using the LDA as the features (this is the global feature in each document) served to enrich the dataset 4 document representation. The experimental results indicated that the B-CNN framework for the 20 newsgroups dataset was slightly better than those of the other methods. We plan to investigate other features relevant to the newsgroups dataset in future research.

Table 7 shows the results for the 20 newsgroups dataset with four classes. We compared the best results obtained from our model with those obtained by Hingmire [80] and Lai [30]. The CNN in this table is described in [29], and we directly quote the results from Lai’s paper. We used the word embedding representation on a general dataset rather than the newsgroups dataset. Unsurprisingly, our result was similar to Lai’s. Our model performed better than the CNN, which indicates that combining the global features of the two models (merged by the DBM model) can increase the performance of the model. Furthermore, this concept is applicable to other fields.

**Table 7:** Classification results (%) on dataset 4 (C = 4).

Model	20 News
ClassifyLDA-EM [80]	93.60%
RCNN [30]	96.49%
CNN [29]	94.79%
B-CNN	95.13%

## 6 Conclusion

This paper presents a new model (B-CNN) for semantic indexing, which is based on the CNN and can learn semantic representations. We tested this model using the task of indexing biomedical abstracts. Our experimental results demonstrate that the low-dimensional representation of the output layer in our framework is more compact and effective compared with a number of alternative methods. We have found that adaptively grouping word2vec categories into (coarse) subsets via clustering is an effective way to remove noise from unbalanced distributed samples. This is especially the case when dealing with a large number of labels and a massive number of biomedicine documents. Our proposed hierarchical indexing structure achieves effective performance, and can be easily extended to other multi-class indexing tasks. However, our model faces the problem of gradients vanishing when the number of categories in the unbalanced samples is extremely large. The long short term memory network can effectively learn features and also obtain strong results on a variety of sequence modeling tasks. Therefore, we plan to introduce this model in our future research.

There are three issues in our future research: 1) how to effectively construct the hierarchical indexing label tree with different dependencies among labels to improve the accuracy of multi-label classification, e.g., label embedding relations (already mentioned in this paper) and co-occurrence relations; 2) how to develop an approach to predict labels with variable lengths in multi-label prediction; and 3) how to extend our approach to apply to image’s text classification [81–83].
